# Skin damage induced by zinc oxide nanoparticles combined with UVB is mediated by activating cell pyroptosis via the NLRP3 inflammasome–autophagy–exosomal pathway

**DOI:** 10.1186/s12989-021-00443-w

**Published:** 2022-01-05

**Authors:** Yu-Ying Chen, Yu-Hsuan Lee, Bour-Jr Wang, Rong-Jane Chen, Ying-Jan Wang

**Affiliations:** 1 Department of Environmental and Occupational Health, College of Medicine, National Cheng Kung University, 138 Sheng-Li Road, Tainan, 70428 Taiwan; 2 Department of Cosmeceutics, China Medical University, Taichung, Taiwan; 3 Department of Cosmetic Science and Institute of Cosmetic Science, Chia Nan University of Pharmacy and Science, Tainan, 71710 Taiwan; 4 Department of Occupational and Environmental Medicine, National Cheng Kung University Hospital, Tainan, 70403 Taiwan; 5 Department of Food Safety/Hygiene and Risk Management, College of Medicine, National Cheng Kung University, 138 Sheng-Li Road, Tainan, 70428 Taiwan; 6 Department of Medical Research, China Medical University Hospital, China Medical University, Taichung, Taiwan

**Keywords:** Zinc oxide nanoparticles, NLRP3 inflammasomes, Pyroptosis, Autophagy, Exosomes

## Abstract

**Background:**

Zinc oxide nanoparticles (ZnONPs) are widely used nanomaterial in personal cosmetics, such as skin creams and sunscreens, due to their whitening properties and strong UV light absorption. However, the safety issues and the hazards of ZnONPs, which can be taken up by the skin and cause skin toxicity, are still unclear. From a chemoprevention point of view, pterostilbene (PT) has been reported to prevent skin damage effectively by its anti-inflammatory and autophagy inducer effect. This study aims to determine the skin toxicity and the potential mechanisms of UVB and ZnONPs exposure and the preventive effect of PT.

**Results:**

The co-exposure of UVB and ZnONPs elicit NLRP3 inflammasome activation and pyroptosis in keratinocytes. Furthermore, exposure to both UVB and ZnONPs also disrupts cellular autophagy, which increases cell exosome release. In vivo UVB and ZnONPs exposure triggers skin toxicity, as indicated by increased histological injury, skin thickness and transepidermal water loss. Notably, the NLRP3 inflammasome-mediated pyroptosis are also activated during exposure. Topical application of pterostilbene attenuates NLRP3 inflammasome activation and pyroptosis by decreasing ROS generation and mitochondrial ROS (mtROS) levels. In addition to its antioxidant effect, PT also reversed autophagy abnormalities by restoring normal autophagic flux and decreasing NLRP3 inflammasome-loaded exosome release.

**Conclusions:**

Our findings reveal that ZnONPs induce skin damage in conjunction with UVB exposure. This process involves an interplay of inflammasomes, pyroptosis, autophagy dysfunction, and exosomes in skin toxicity. PT alleviates skin inflammation by regulating the inflammasome–autophagy–exosome pathway, a finding which could prove valuable when further evaluating ZnONPs effects for cosmetic applications.

**Supplementary Information:**

The online version contains supplementary material available at 10.1186/s12989-021-00443-w.

## Background

The widespread application of nanotechnology, especially the increasing number of applications for nanomaterials in the therapeutic and cosmetic industries, has considerably increased their biosafety concerns [1, 2]. Zinc oxide nanoparticles (ZnONPs) are widely used nanomaterial in personal cosmetics, such as skin creams and sunscreens, due to their whitening properties and strong ultra violet (UV) light absorption [3]. However, it is a long-standing argument about the safety concern regarding the use of ZnONPs, which can be taken up by skin and cause skin toxicity. Several studies indicated that ZnONPs might not penetrate skin. Leite-Silva et al. demonstrated that topically applied ZnONPs did not penetrate the intact epidermis or barrier–impaired skin of volunteers and exhibited no harmful effects [4, 5]. However, Holmes et al. indicated that although topically applied ZnONPs do not penetrate the epidermis, their hydrolysis increases the levels of zinc ion in the stratum corneum, which then accumulates in the epidermis, when introduced into systemic circulation, the zinc ion may induce toxicity [6]. On the other hand, reports have demonstrated that ZnONPs can penetrate the skin in some situation, such as UVB-damaged and allergic skin models [6–10]. Thus, a damaged stratum corneum may allow ZnONPs to contact keratinocytes, leading to potential hazardous and toxicological consequences. The well-known toxicity induced by ZnONPs is predominantly mediated by the formation of reactive oxygen species (ROS) in the presence of light excitation [11]. Excessive ROS generation may damage mitochondria, which subsequently leads to inflammasome activation and cell death [12–14].

Inflammasomes are newly recognized as crucial players in the innate immune response, among which the most characterized pathway is the NLRP3 inflammasome [13]. Activation of NLRP3 can lead to caspase-1-dependent secretion of interleukin-lβ (IL-1β) and IL-18, triggering a process of inflammation-related cell death named pyroptosis. Indeed, pyroptosis is a rapid and inflammatory form of lytic programmed cell death [14, 15]. To date, gasdermin D (GSDMD) has been identified as the pivotal mediator of pyroptosis. Cell death by pyroptosis is first initiated when activated caspase-1 cleaves GSDMD, accompanied by a series steps including membrane pore formation, fluid influx, cell swelling, plasma membrane rupture and eventually leakage of cellular contents [16–18]. The induction of pyroptotic cell death by ZnONPs has never been reported before; thus, we attempted to determine whether activation of NLRP3 and the resulting pyroptosis are involved in the ZnONP-induced cell death of keratinocytes.

The molecular crosstalk between inflammasomes and autophagy, a lysosome-dependent catabolic process for degradation of proteins and organelles, is the focus of an emerging field of research that is important for the understanding of homeostasis in multicellular organisms of a variety of pathological conditions, including skin disorders [19]. A growing number of reports have demonstrated that NPs can induce autophagy activation, and the activated autophagy plays dual roles of both cellular defense and cytotoxicity [20–22]. Although autophagy usually plays a crucial role in cell survival, autophagy dysfunction, a phenomenon in which the cell fails to activate the lysosomal degradation pathway, has been demonstrated by our group and others as an important nanotoxicity pathway in many types of nanomaterials and biological models [21, 23]. In addition, autophagy also possesses the ability to decrease inflammation by suppressing the NLRP3 inflammasome [24]. Thus, autophagy dysfunction causes the accumulation of damaged organelles and enhances ROS generation and NLRP3 complex assembly, and could be a novel mechanism modulating ZnONP-induced inflammatory and cytotoxic effects.

Regarding the crosstalk between inflammasomes and autophagy, exosomes might serve as a bridge under stress conditions such as ZnONPs exposure [25]. However, the detailed mechanisms remain unclear. Both autophagy and exosome release are responsible for eliminating unwanted proteins in which either route may compensate for a deficiency in the other [26, 27]. In addition, inflammasomes can also serve as regulators of protein secretion by exosomes to alert and guide neighboring cells, while cells were exposed to danger signals [28]. Moreover, inflammasome-derived exosomes are preferentially taken up by neighboring immune cells, leading to NLRP3 assembly and caspase-1/IL-1β processing and triggering pyroptosis [29, 30]. Thus, exosomes could possess an important impact on disease by mediating inflammasome and autophagy.

From a chemoprevention point of view, topical application of pterostilbene (PT), a natural phytoalexin, has been reported to protect hairless mice from UVB-induced skin damage effectively [31]. Antioxidative and anti-inflammatory activity of PT is believed to underlie its positive health effects prevent skin disease [32, 33]. Studies also showed that PT has the therapeutic effect in many diseases owing to its autophagy inducing effect [34, 35]. For example, our pervious study showed PT could effectively prevent chronic kidney disease (CKD) via autophagy to restraining TGF-β mediated NLRP3 inflammasome activation [35]. Therefore, one of the purposes of our current study was to investigate the possible effects of PT in keratinocytes in vitro and in mouse skin in vivo treated with ZnONPs alone or in combination with UVB and to explore the involved signaling regulators.

In this study, we investigated the role of NLRP3 inflammasome-dependent pyroptosis, autophagy and exosomes in UVB combine ZnONPs induced skin-toxicity and the preventive effect of PT. To the best of our knowledge, this is the first study demonstrating the detailed underlying molecular mechanisms triggered by ZnONPs in combination with UVB in skin.

## Results

### Physical–chemical characterization and keratinocyte toxicity of ZnONPs

**Fig. 1:**
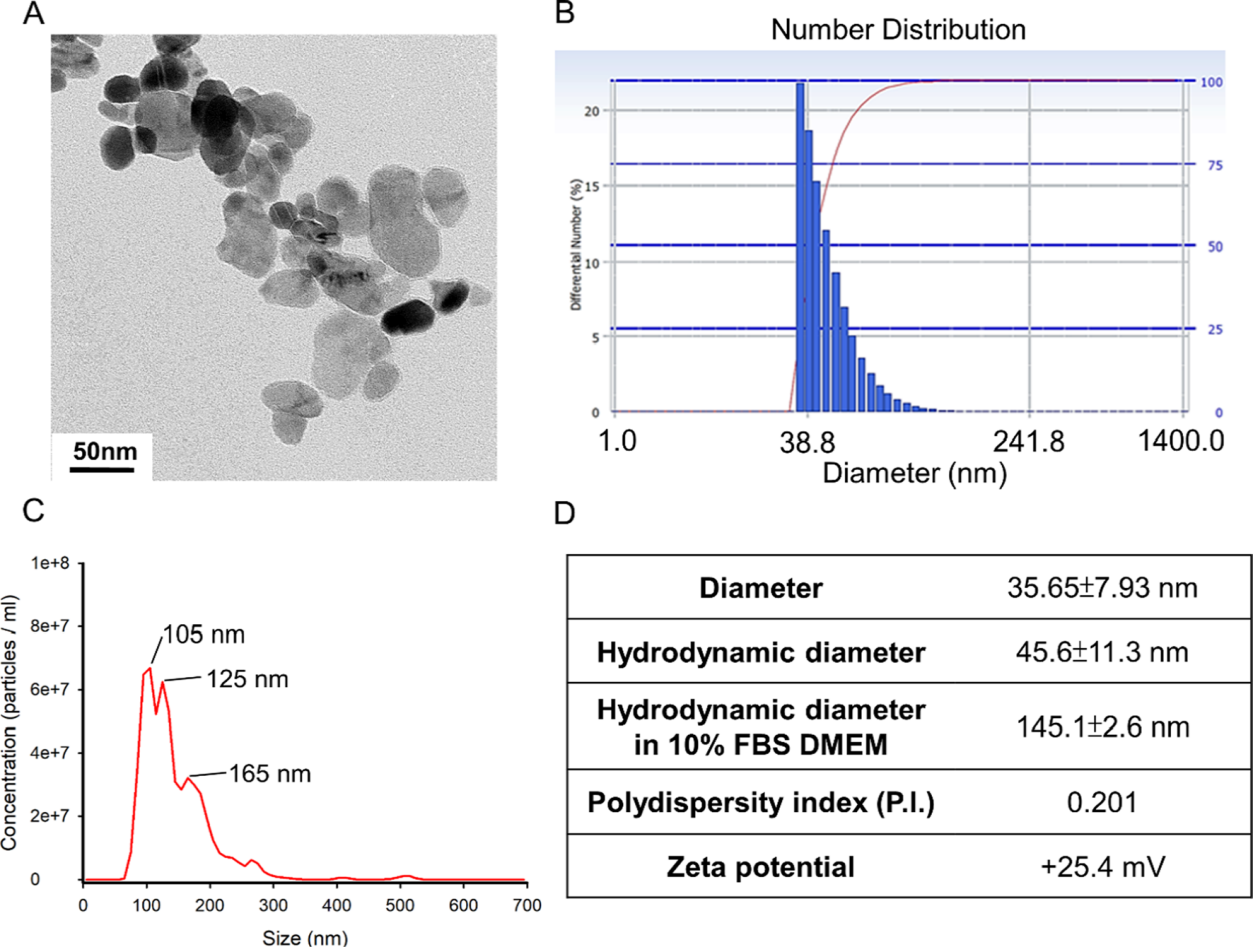
Characterization of ZnONPs. **A** The shape and size of ZnONPs used in this study were determined by transmission electron microscopy. **B** The hydrodynamic size of ZnONPs in distilled water were characterized by dynamic light scattering (DLS). **C** The hydrodynamic size of ZnONPs in complete culture medium (DMEM with 10% FBS) were characterized by nanoparticle tracking analysis (NTA). **D** Characterization of the diameter, hydrodynamic diameter in distilled water and complete medium, polydispersity index (P.I.) and zeta potential of ZnONPs. Values are presented as the mean ± standard deviation averaged over three replicates

**Fig. 2:**
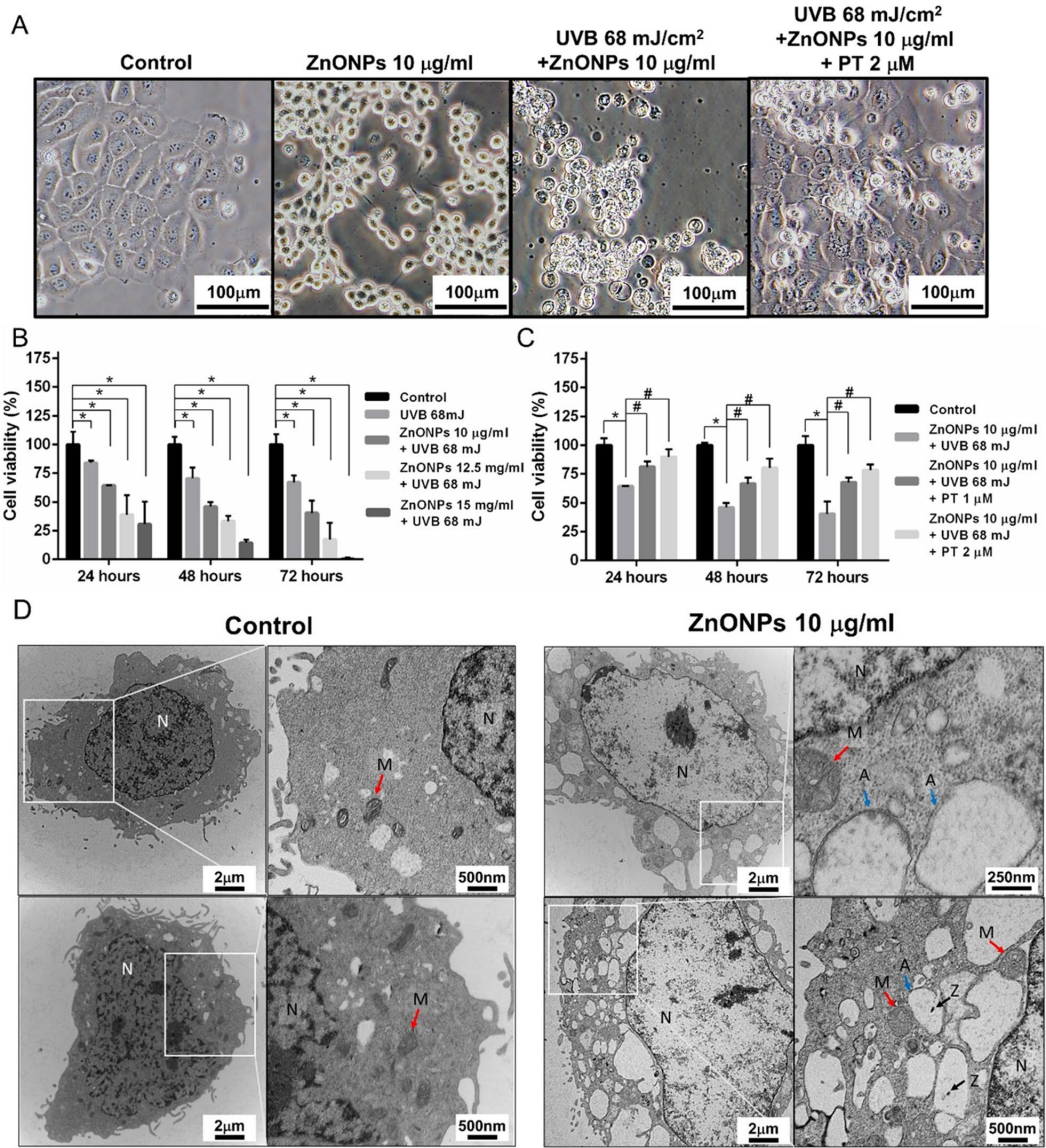
Morphology change and viability of HaCaT cells treated with ZnONPs and UVB. **A** Morphological changes after treatment with ZnONPs (10 μg/mL), UVB + ZnONPs (68 mJ/cm^2^ + 10 μg/mL) and UVB + ZnONPs + PT (2 μM). **B** Cell viability assay showing the dose-dependent cytotoxicity of combined treatment with ZnONPs (0–15 μg/mL) and UVB (68 mJ/cm^2^). **C** PT treatment (0–2 μM) followed by ZnONPs (10 μg/mL) + UVB (68 mJ/cm^2^) significantly increased the viability of HaCaT cells. **D** TEM image of HaCaT cells after ZnONPs (10 μg/mL) treatment for 3 h. The autophagosome double-membrane structures (blue arrow) engulfed ZnONPs (black arrow), the mitochondrial outer membranes were swollen, and the inner cristae were highly degenerated (red arrow). Values are presented as the mean ± SD (n = 3). *p < 0.05, control group versus treatment groups. ^#^*p* < 0.05, the UVB + ZnONPs groups versus the UVB + ZnONPs + PT groups. *Abbreviations N* nucleus, *A* autophagosome, *Z* ZnONPs, *M* mitochondria

This study used amine-modified zinc oxide nanoparticles (NH_2_-ZnONPs). The morphology and structure of ZnONPs were observed by transmission electron microscopy (TEM) (Fig. 1A). The average hydrodynamic diameter and polydispersion of the ZnONPs were measured by using dynamic light scattering (DLS) (Fig. 1B), which showed that the mean diameter of ZnONPs was approximately 35.65 ± 7.93 nm. The DLS data revealed that the hydrodynamic size of ZnONPs was 45.6 ± 11.3 nm in aqueous solution and that ZnONPs had a positive surface charge of 25.4 mV (Fig. 1D). When the ZnONPs were dispersed in culture medium (DMEM with 10% fetal bovine serum), the mean particle size was approximately threefold the primary size (145.1 ± 2.6 nm) (Fig. 1C, D), which could be taken up by HaCaT cells, as demonstrated by fluorescence microscopy analysis of HaCaT cells after exposure to 10 μg/mL R6G-ZnONPs (Additional file 1: Figure 1A). In addition, the results of flow cytometry analysis showed that the cellular FSC and SSC signals increased, indicating that the internal complexity was increased after ZnONPs exposure (Additional file 1: Figure 1B). Morphologically, HaCaT cells showed swelling and spherical shapes after exposure to ZnONPs and UVB. In contrast, the addition of PT significantly protected HaCaT cells against these morphological changes (Fig. 2A). Cell viability studies were undertaken, with trypan blue assay used to obtain the toxicological profiling of UVB when using ZnONPs and PT alone or in combination to treat HaCaT cells. Cells were first tested with treatments of only ZnONPs or PT by using a series of dosages. The HaCaT cells showed significant dose- and time-dependent cytotoxicity in the ZnONP treatment of 7.5–17.5 μg/mL (Additional file 1: Figure 2A). Regarding the effects of PT, 2 μM PT treatment yielded no significant toxicity at any time point (24, 48, or 72 h), whereas 3 μM PT yielded significant cytotoxicity at 24 h (Additional file 1: Figure 2B). Therefore, we applied 2 μM PT in our further experiments. After the cells were exposed to ZnONPs and UVB for 24 h, a dose-dependent decrease in cell viability was observed (Fig. 2B). As expected, PT (2 μM) significantly protected cells against ZnONPs and UVB-induced cell death. These findings indicated that ZnONP and UVB exposure induced severe cytotoxicity and that PT had the ability to rescue cell death (Fig. 2C). For in-depth investigation of the mechanisms, we applied ZnONPs at a 10 μg/mL concentration combined with UVB (68 mJ) in our further experiments.

### Mitochondrial damage induced by ZnONPs and UVB triggers NLRP3 inflammasome activation and pyroptosis

**Fig. 3:**
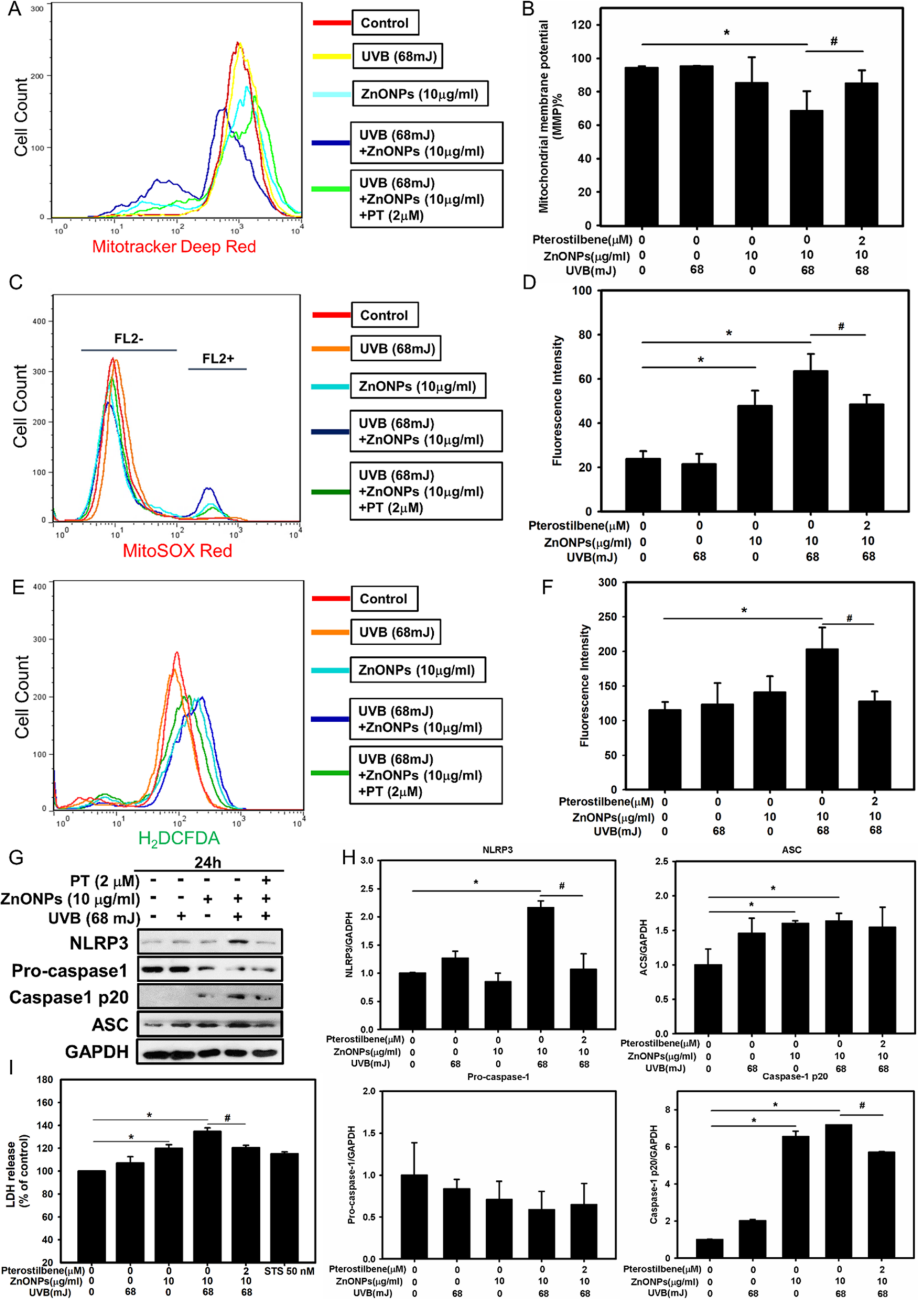
ZnONP- and UVB-induced Mitochondrial damage and NLRP3 inflammasome activation in HaCaT cells. **A** Mitotracker Deep Red was employed to examine ZnONP-induced mitochondrial membrane potential (MMP) loss via flow cytometry after 24 h exposure. **B** Histograms represent the percentage of MMP loss. **C** mtROS generation were examined by flow cytometry in HaCaT cells after ZnONPs and UVB treatment in 3 h. **D** Histograms represent the fluorescence intensity of mtROS. **E** ROS generation were examined by flow cytometry in HaCaT cells after ZnONPs and UVB treatment in 3 h. **F** Histograms represent the fluorescence intensity of ROS. **G**, **H** Western blot analysis of the levels of the NLRP3 inflammasome proteins NLRP3, ASC, pro-caspase-1 and cleaved caspase-1 in HaCaT cells. GAPDH was used as a loading control. **I** LDH release in ZnONP-treated HaCaT cells. Values are presented as the mean ± SD (n = 3). *p < 0.05, control group versus treatment groups. ^#^*p* < 0.05, the UVB + ZnONPs groups versus the UVB + ZnONPs + PT groups

Mitochondrial homeostasis plays a vital role in the cellular response to stress. Dysfunction of mitochondria and the release of mtROS in cell is a key upstream event involved in NLRP3 activation [16]. We first investigated the effects of ZnONPs and UVB on mitochondrial homeostasis by using TEM to observe the ultrastructure of mitochondria. After treatment with ZnONPs, the mitochondria showed swelling of the external membrane, and the mitochondrial cristae were highly degenerated, indicating severe mitochondrial damage (Fig. 2D). Second, the mitochondrial membrane potential (MMP) results showed that MMP distinctly decreased after ZnONPs (10 μg/mL) and UVB (68 mJ/cm^2^) treatment for 24 h (Fig. 3A, B), and PT (2 μM) significantly inhibited the MMP decrease caused by ZnONPs and UVB exposure. Third, we employed MitoSox, a specific mitochondrial ROS probe, to further demonstrate that the administration of ZnONPs and UVB increased mtROS in HaCaT cells in 3 h, and PT treatment also inhibited mtROS generation (Fig. 3C, D). Last, we tested whether ZnONPs induce total ROS generation. As shown in Fig. 3E, F, the intracellular ROS level significantly increased with ZnONPs and UVB treatment in 3 h, and PT treatment attenuated ROS generation in cells. These results indicated that ZnONPs and UVB exposure could induced mitochondrial damage and ROS production.

**Fig. 4:**
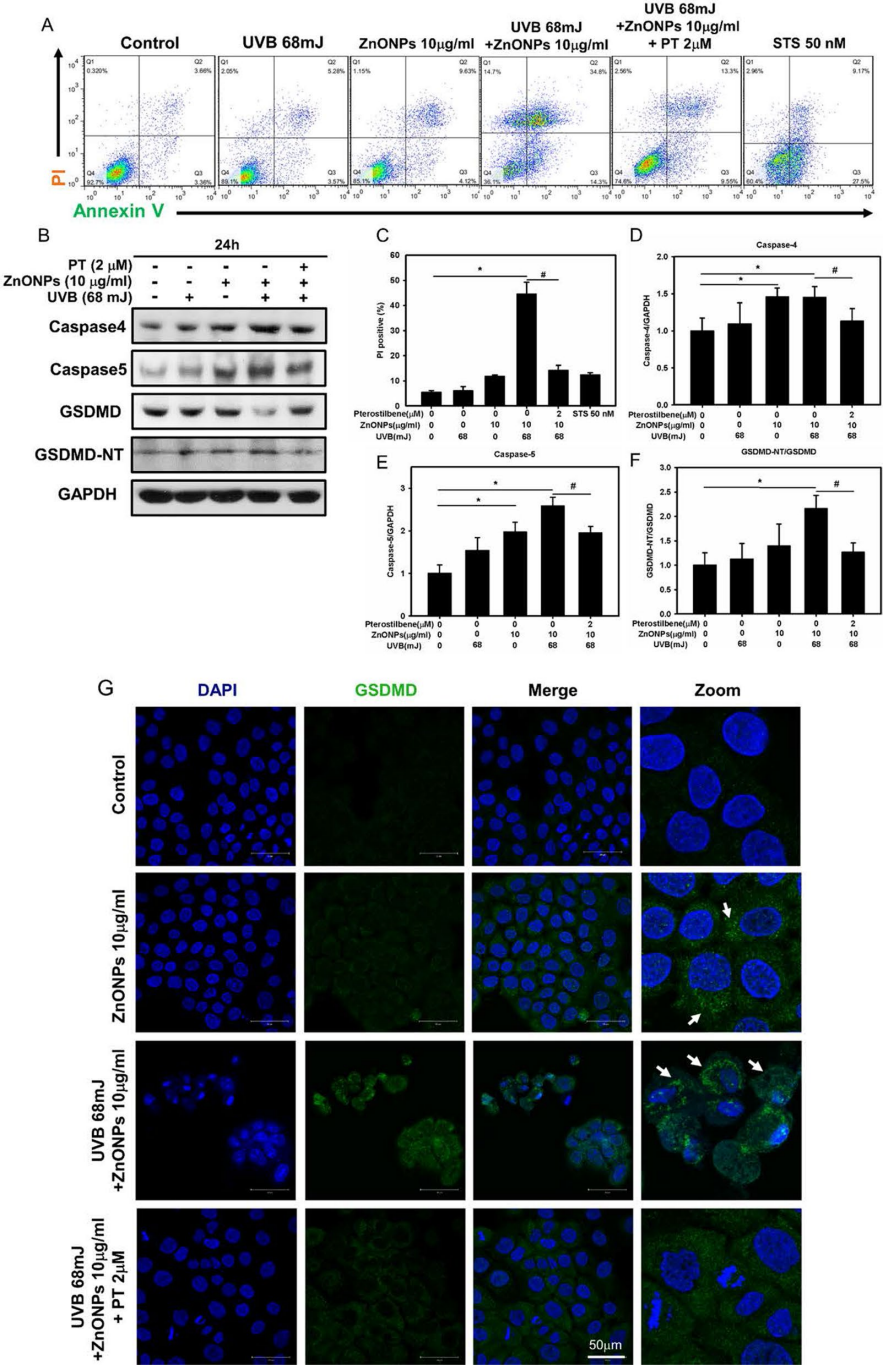
Effect of ZnONP- and UVB-triggered HaCaT cell pyroptosis. **A** Annexin V and PI were employed to examine ZnONP induced HaCaT cell pyroptosis via flow cytometry. **C** Histograms represent the percentage of Q1 + Q2 regions, indicating PI positive cells. **B**–**F** The levels of the pyroptosis proteins caspase 4, caspase 5, GSDMD and cleaved GSDMD-NT in HaCaT cells were detected by Western blotting, and GAPDH was used as a loading control. **G** HaCaT cell immunofluorescence staining with an anti-GSDMD antibody after a 24 h treatment with ZnONPs (10 μg/mL), UVB (68 mJ/cm^2^) + ZnONPs (10 μg/mL) or UVB (68 mJ/cm^2^) + ZnONPs (10 μg/mL) + PT (2 μM). Arrows indicate GSDMD puncta. Values are presented as the mean ± SD (n = 3). *p < 0.05, control group versus treatment groups. ^#^*p* < 0.05, the UVB + ZnONPs groups versus the UVB + ZnONPs + PT groups

To determine whether the NLRP3 inflammasome was activated in HaCaT cells after ZnONPs and UVB treatment, we detected the expression of the NLRP3 inflammasome proteins NLRP3, ASC, pro-caspase-1 and cleaved caspase-1. As shown in Fig. 3G, H, the levels of NLRP3, ASC, and cleaved caspase-1 were increased in ZnONP- and UVB-treated HaCaT cells, indicating activation of the NLRP3 inflammasome. Based on the observation that caspase-1 was activated in HaCaT cells, we hypothesized that pyroptosis could have occurred. Pyroptosis is dependent on GSDMD activation and will cause pore formation on the cell membrane [16, 36]. To test this hypothesis, we first employed LDH and PI/Annexin V assays to evaluate cellular rupture caused by pore formation on the cell membrane [37, 38]. In addition, to further distinguish the pyroptosis membrane rupture from classic apoptosis, we applied staurosporine (STS, 50 nM), a well-known apoptosis inducer, to serve as a negative control of pyroptosis. The results showed that both LDH release and PI signals increased significantly in ZnONPs and UVB group compared to the control and the STS groups (Figs. 3I, 4A, C). Then, we measured the protein expression of pyroptosis markers in ZnONP- and UVB-treated HaCaT cells and found increased expression of pro-GSDMD and GSDMD-NT (Fig. 4B, D–F). Interestingly, the increase in inflammasome and pyroptosis proteins induced by ZnONPs and UVB was reversed by PT (Fig. 4B, D–F). Moreover, we applied immunofluorescence staining with the GSDMD antibody, and the results further confirmed the abovementioned findings that GSDMD expression was increased after ZnONP- and UVB-treatment. The PT treatment inhibited GSDMD expression (Fig. 4G). To clarify the link between the NLRP3 inflammasome and pyroptosis, we used sh-*caspase1* to knock down the expression of the key NLRP3 inflammasome effector caspase-1. Compared with nontarget vesicle control, *sh-caspase1* resulted in a specific and significant reduction in the protein level of caspase-1. After caspase-1 inhibition, the PI/Annexin V assay showed a decrease in cell death (Additional file 1: Figure 3A, C). In addition, the pyroptosis proteins caspase 4, caspase 5 and GSDMD were also decreased after caspase-1 silencing (Additional file 1: Figure 3B, D–F). Collectively, these results demonstrated that ZnONPs and UVB triggered NLRP3 inflammasome and pyroptosis activation in keratinocytes and that PT treatment attenuated NLRP3 inflammasome and pyroptosis activation.

### ROS play pivotal roles in ZnONP- and UVB-induced NLRP3 inflammasome activation and pyroptosis

**Fig. 5:**
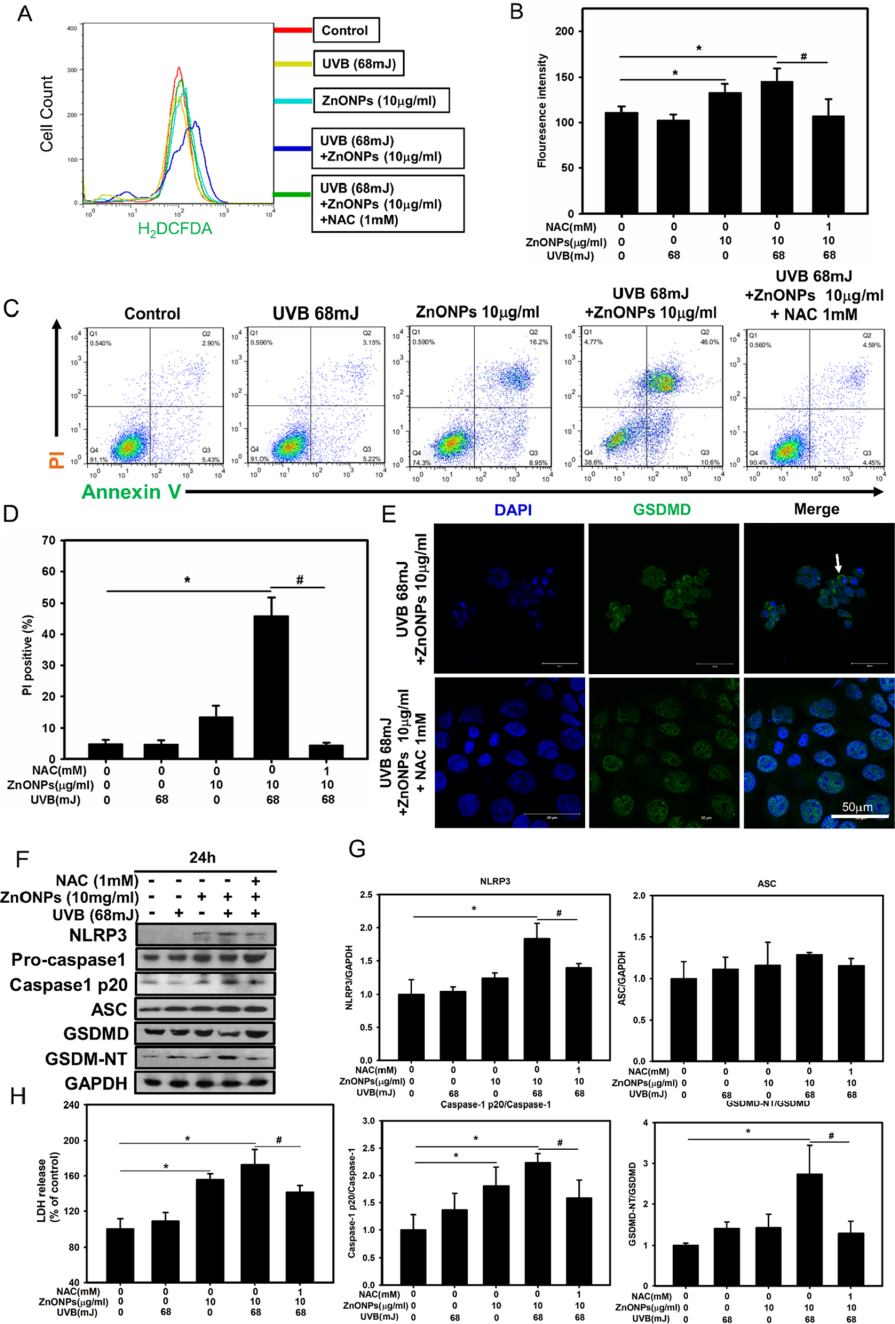
ZnONP-induced NLRP3 activation and pyroptosis are mediated by ROS in HaCaT cells. **A** Inhibitory effect of NAC on the ZnONP- and UVB-elicited production of ROS. **B** Histograms represent the fluorescence intensity of ROS. **C** Annexin V and PI were employed to examine ZnONP-induced HaCaT cell pyroptosis via flow cytometry. **D** Histograms represent the percentage of Q1 + Q2 regions, indicating PI positive cells. **E** Immunofluorescence staining with an anti-GSDMD antibody of HaCaT cells treated with UVB (68 mJ/cm^2^) + ZnONPs (10 μg/mL), UVB (68 mJ/cm^2^) + ZnONPs (10 μg/mL) + NAC (1 mM) for 24 h. Arrow indicate GSDMD puncta. **F**, **G** Western blot analysis of the effects of NAC on the ZnONP-induced NLRP3 inflammasome and pyroptosis proteins NLRP3, caspase-1, cleaved caspase-1, ASC, GSDMD and cleaved GSDMD-NT in HaCaT cells. **H** LDH release in ZnONP-treated HaCaT cells treated with NAC. Values are presented as the mean ± SD (n = 3). *p < 0.05, control group versus treatment groups. ^#^*p* < 0.05, the UVB + ZnONPs groups versus the UVB + ZnONPs + NAC groups

ROS generation is one of the mechanisms by which the NLRP3 inflammasome can be activated [1]. In order to assess the role of ROS in NLRP3 inflammasome activation, HaCaT cells were pretreated with NAC (1 mM). NAC pretreatment significantly decreased the intracellular ROS level and alleviated cell death compared to ZnONPs and UVB treatment alone in 3 h (Fig. 5A, B). After NAC treatment, the PI/Annexin V assay showed a decrease in cell death (Fig. 5C, D) and LDH release was also decrease significantly (Fig. 5H). Moreover, immunoblotting assays showed that NAC reduced the expression of NLRP3, ASC, pro-caspase-1, cleaved caspase-1 and GSDMD in HaCaT cells after treatment with ZnONPs combined with UVB (Fig. 5F, G). Immunofluorescence staining also showed that NAC reduced the number of puncta with positive staining for the pyroptosis protein GSDMD (Fig. 5E). These results indicated that ROS played a crucial role in ZnONP- and UVB-activated NLRP3 inflammasome and pyroptosis.

### ZnONP- and UVB-triggered autophagy dysfunction is inhibited by PT in keratinocytes

**Fig. 6:**
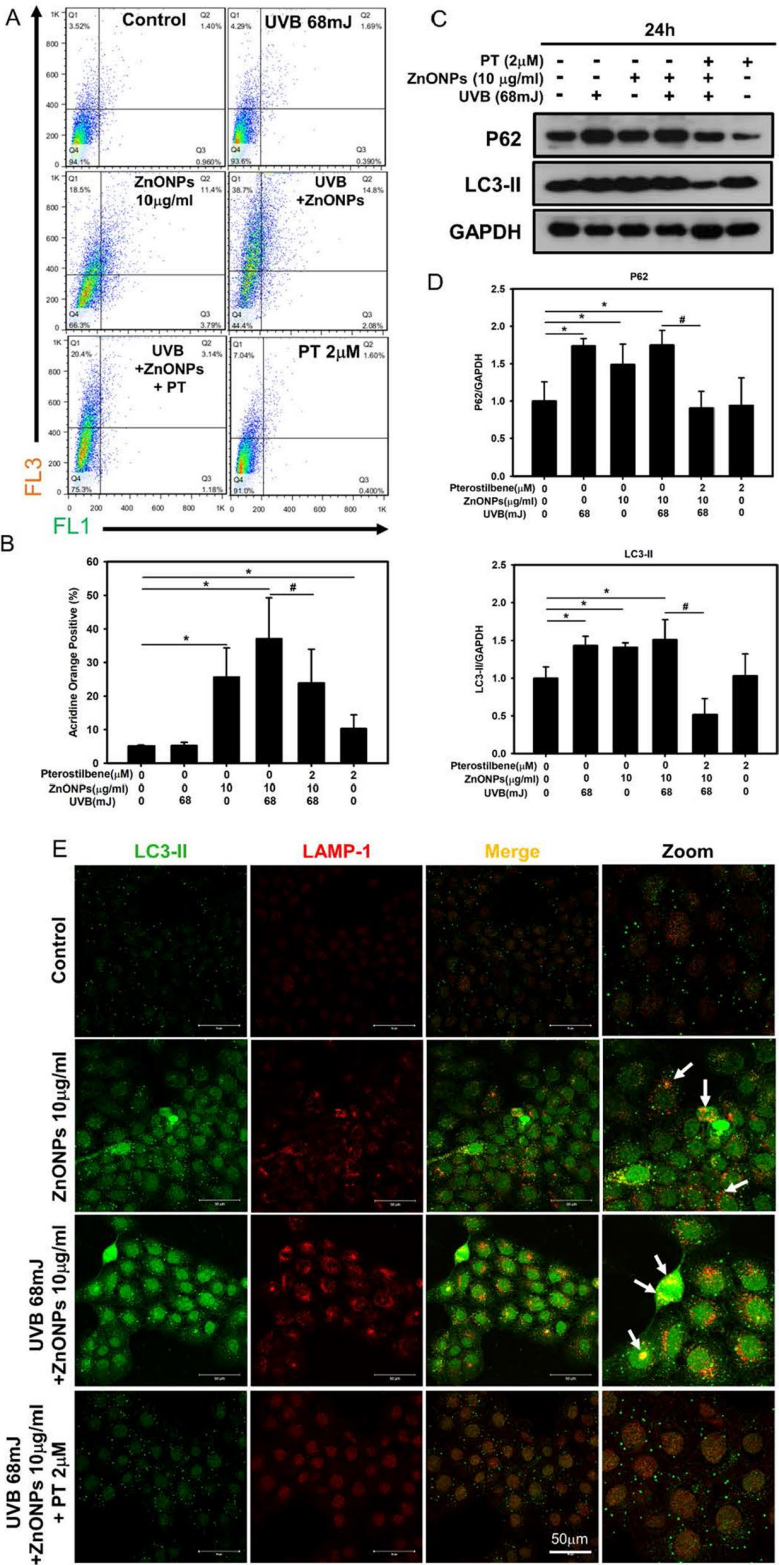
ZnONP-induced autophagy dysfunction in HaCaT cells. **A** Acridine orange staining was employed to examine the number of acidic vesicular organelles (AVOs), such as autophagolysosomes, in cells. Flow cytometry analysis demonstrated the effect of PT against ZnONP- and UVB-induced keratinocyte autophagolysosomes. The FL3 positive region (Q1 + Q2 regions) indicated the AO positive staining. **B** Histograms represent the percentage of acridine orange positive cells. **C**, **D** Western blot analysis of the effect of ZnONPs and UVB on the autophagy proteins LC3B and p62 in HaCaT cells. **E** Immunofluorescence staining with anti-LC3B/anti-LAMP1 antibodies in HaCaT cells treated with ZnONPs (10 μg/mL), UVB (68 mJ/cm^2^) + ZnONPs (10 μg/mL) or UVB (68 mJ/cm^2^) + ZnONPs (10 μg/mL) + PT (2 μM) for 24 h. Arrows indicate LC3B/LAMP1 co-localized puncta. Values are presented as the mean ± SD (n = 3). *p < 0.05, control group versus treatment groups. ^#^*p* < 0.05, the UVB + ZnONPs groups versus the UVB + ZnONPs + PT groups

Recent studies have highlighted the cross link between inflammasome and autophagy. Autophagy activation can limit NLRP3 inflammasome through the intracellular degradation system of autophagy [22]. On the other hands, autophagy dysfunction is considered an early indicator of nanomaterial interactions with cells, and autophagosome accumulation could serve as a general phenotype induced by nanoparticles [17]. As shown in Fig. 2D, the subcellular organelles were morphologically normal in untreated control cells with rare autophagic vacuoles, while a large number of double-membrane autophagosomes were obviously observed in ZnONP-treated HaCaT cells. Moreover, autophagosome-engulfed ZnONPs were also observed by TEM. These data show autophagosome accumulation under ZnONPs treatment. Autophagic flux was assessed using acridine orange (AO) staining (Fig. 6A). AO staining was measured the number of acidic vesicular organelles (AVOs), such as autophagolysosomes in cells [39]. Autophagy activation can be assessed by measuring the change in intracellular AO fluorescence. The intracellular AO fluorescence level significantly increased with ZnONPs and UVB treatment, and PT treatment significantly decreased the AO fluorescence intensity (Fig. 6B). We then used Western blotting to analyze the autophagic flux proteins LC3B and p62 and showed that the expression of LC3B-II and p62 was increased under ZnONPs and UVB treatment, whereas PT treatment decreased LC3B and p62 expression (Fig. 6D). These results imply that ZnONPs and UVB might have blocked autophagic flux, resulting in autophagy dysfunction, and PT treatment reversed this dysfunction. To confirm the blockage of autophagic flux by ZnONPs and UVB treatment, we used LAMP-1 (lysosome marker) and LC3B (autophagosome marker) double immunofluorescence staining to monitor co-localization of lysosome and autophagosome in autophagic flux. The ZnONPs and UVB treatment group showed separate green and red fluorescence, indicating that lysosome and autophagosome were unable to fuse and autophagic flux was blocked. The PT treatment group showed significant yellow fluorescence, which indicated the successful fusion of autolysosomes, showing that PT treatment restored the blocked autophagic flux (Fig. 6E).

### Exosomes mediate ZnONP- and UVB-induced cell-to-cell transmission of NLRP3 inflammasome components

**Fig. 7:**
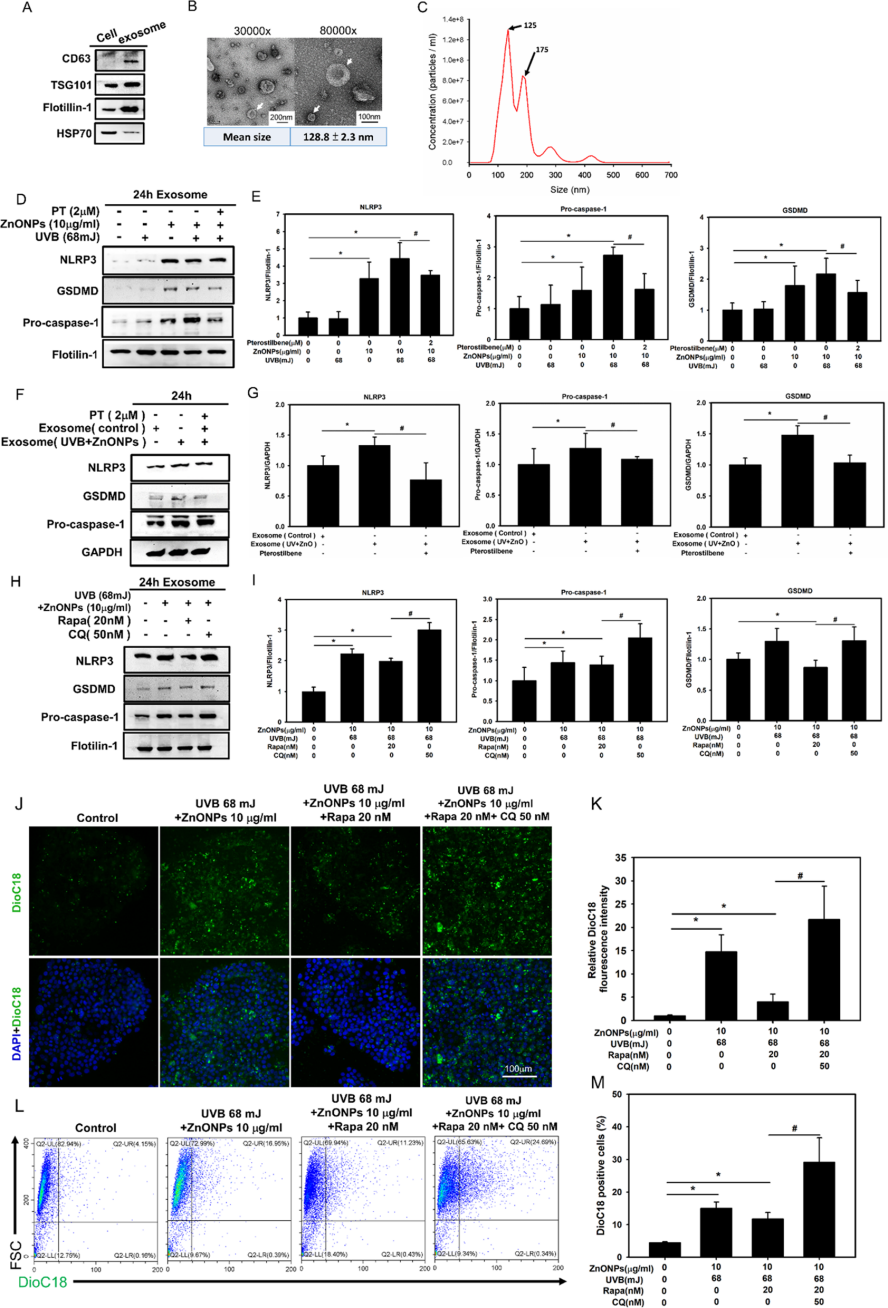
Exosomes release NLRP3 inflammasome complex propagates ZnONP- and UVB-induced keratinocytes inflammation. **A** Western blot analysis of isolated exosomes. The presence of canonical exosome proteins and the HSP70 decreasing indicate a pure exosome preparation. **B** TEM imaging of exosomes, arrows indicated the exosomes with cup-shaped morphology. **C** NTA analysis demonstrated a size distribution of particles that was consistent with the size range of exosomes. **D**, **E** Western blot analysis of exosomes isolated from the medium of HaCaT cells treated with ZnONPs and UVB. The NLRP3 inflammasome and pyroptosis proteins NLRP3, pro-caspase-1 and GSDMD were detected, Flotillin-1 was used as a housekeeping protein (loading control). **F**, **G** Western blot analysis of NLRP3, caspase-1 and GSDMD expression in HaCaT cells after treatment with PT and exosomes isolated from control cells or cells exposed to ZnONPs and UVB. **H**, **I** Western blot analysis of exosomes isolated from HaCaT cells after treatment with rapamycin (autophagy inducer), chloroquine (autophagy inhibitor), ZnONPs and UVB. The NLRP3 inflammasome and pyroptosis proteins NLRP3, caspase-1 and GSDMD were detected, Flotillin-1 was used as a housekeeping protein (loading control). **J** A Transwell culture system was used to mimic the cellular milieu. HaCaT cells were exposed to foreign DioC18-labeled exosomes from donor HaCaT cells that were exposed to ZnONPs and UVB with or without Rapa or Rapa + CQ treatment. **K** Histograms represent the relative fluorescence intensity of DioC18 staining. **L** DioC18-labeled exosomes propagation was measured via flow cytometry. DioC18 positive HaCaT cells (Q2-UR + LR regions) indicate were exposed to foreign DioC18-labeled exosomes from donor HaCaT cells that were exposed to ZnONPs and UVB with or without Rapa or Rapa + CQ treatment. **M** Histograms represent the DioC18 positive cells. Values are presented as the mean ± SD (n = 3). *p < 0.05, the control group versus the treatment groups. ^#^*p* < 0.05, the UVB + ZnONPs group versus the UVB + ZnONPs + PT group, Exosome (UVB + ZnONPs) group versus Exosome (UVB + ZnONPs) + PT group, UVB + ZnONPs + Rapa group versus UVB + ZnONPs + CQ group, UVB + ZnONPs + Rapa group versus UVB + ZnONPs + Rapa + CQ group

Our previous study indicated that ZnONPs and UVB induced the NLRP3 inflammasome in HaCaT cells [40]. A recent study indicated that cells can propagate exosomes loading with NLRP3 inflammasome protein complex via and affect other cellular behaviors [41]. Thus, we hypothesized that ZnONPs and UVB may promote NLRP3 inflammasome and pyroptosis propagation by inflammasome protein loading in exosomes. To determine the importance of exosome cargo for inflammasome propagation, we used ultracentrifugation to isolate exosomes from the medium of HaCaT cells treated with ZnONPs and UVB. Figure 7A demonstrates the presence of the canonical exosome proteins CD63, TSG101 and Flotillin-1 and the decrease of the intracellular protein HSP70, which are typical exosome features. The TEM images show that the exosomes exhibited a cup-shaped morphology (Fig. 7B). NTA analysis demonstrated a size distribution of particles between approximately 125 and 175 nm, which is consistent with the size range of exosomes (Fig. 7C). Western blot analysis was used to determine whether exosomes play a role in inflammasome propagation, and the results showed that ZnONPs and UVB exposure enhanced the expression of exosome loaded NLRP3 inflammasome proteins NLRP3, caspase-1 and the pyroptosis protein GSDMD when normalized with flotillin-1 (housekeeping protein) (Fig. 7D, E).

To test whether exosomes transfer NLRP3 inflammasome and pyroptosis proteins between cells, HaCaT cells were treated with ZnONPs and UVB for 24 h, and then we collected exosomes from the medium. When we exposed cells to exosomes from either ZnONP- and UVB-treated HaCaT cells or untreated cells, Western blot analysis revealed significantly higher NLRP3, caspase-1 and GSDMD protein expression in the former group than in the latter group (Fig. 7F, G). This increase in NLRP3, caspase-1 and GSDMD was not observed when we added 0.1% Triton X-100 to the exosome solution to denature exosome function before the treatment (Additional file 1: Figure 4A). Collectively, these findings suggest that ZnONPs and UVB exposure induced cell-to-cell transmission of NLRP3 inflammasome and pyroptosis proteins via exosomes, which further propagated inflammasome and pyroptosis activation. Treatment with PT decreased the release of inflammasome- and pyroptosis-loaded exosomes.

### Autophagy dysfunction increases exosome release in keratinocytes

Recent studies indicated autophagy is a key regulator of exosomal biogenesis. Autophagosomes fuses with multivesicular bodies (MVBs), a late endosome organelle, to form hybrid organelles termed amphisomes, which can subsequently fuse with lysosomes for content degradation [26, 42]. We hypothesized that the ZnONP- and UVB-induced autophagy dysfunction might increase cellular exosome release. To test this hypothesis, we used the autophagy inducer rapamycin and the autophagy inhibitor chloroquine (CQ) in combination with ZnONPs and UVB exposure. Western blot analysis showed that treatment with rapamycin in combination with ZnONPs and UVB decreased the relative expression of exosome loaded NLRP3 inflammasome proteins NLRP3, caspase-1 and the pyroptosis protein GSDMD when normalized with flotillin-1 (housekeeping protein) (Fig. 7H, I). In contrast, when we blocked autophagic flux by using CQ, the exosomal release of NLRP3, caspase-1, and the pyroptosis protein GSDMD was significantly increased (Fig. 7H, I). We further analyzed the exosome concentration changes by using Transwell assays, and we established coculture systems that mimicked the cellular milieu wherein HaCaT cells were exposed to exosomes on the apical surface. The results demonstrated that an increasing number of HaCaT cell-propagated exosomes were internalized by other HaCaT cells when the upper chamber of the Transwell was exposed to ZnONPs and UVB. Pretreatment with rapamycin significantly decreased exosome release compared to ZnONPs and UVB exposure alone. In contrast, CQ treatment significantly increased exosome release (Fig. 7K). These data indicated that treatment with CQ, an autophagy inhibitor, enhanced ZnONP- and UVB-induced autophagy dysfunction and autophagosome accumulation and enhanced exosome release from HaCaT cells. The autophagy inducer rapamycin decreased exosome release.

### PT inhibits ZnONP- and UVB-induced activation of the NLRP3 inflammasome and activation of caspase-1 in a mouse skin model

**Fig. 8:**
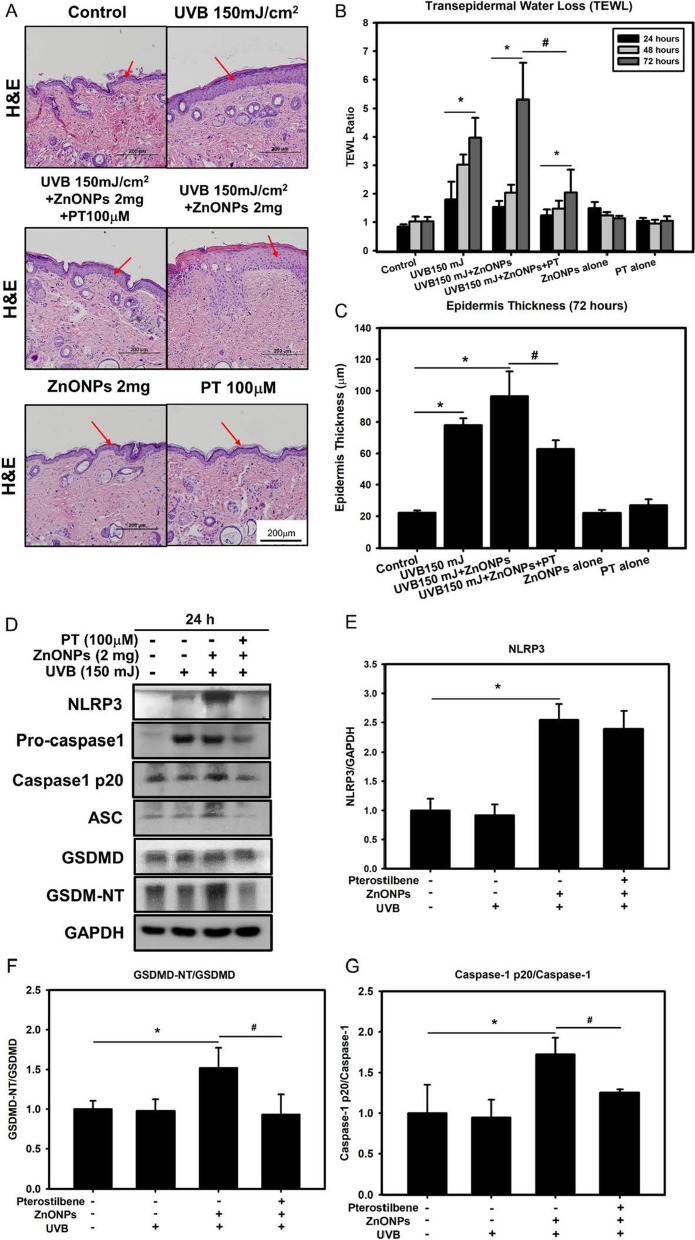
ZnONPs and UVB activate the NLRP3 inflammasome-induced pyroptosis in the skin. **A** H&E histology of mouse skin at 72 h after the acute inflammation test with ZnONPs and UVB showed dermal swelling and inflammatory cell infiltration (red arrows). ZnONPs and UVB treatment significantly increased the dermal thickness, as shown by H&E staining **A**, **C**. PT treatment significantly suppressed ZnONP- and UVB-induced increases in dermal thickness (scale bar represents 200 μm). Transepidermal water loss (TEWL) in SKH:HR-1 mice treated with control (untreated), ZnONPs (2 mg), UVB (150 mJ/cm^2^) and PT (100 µM) alone or in different combinations. **B** Epidermis thickness in mice treated with control (untreated), ZnONPs (2 mg), UVB (150 mJ/cm^2^) and PT (100 µM) alone or in different combinations. **D**–**G** The expression of the NLRP3 inflammasome and pyroptosis proteins NLRP3, ASC, caspase-1, cleaved caspase-1 and GSDMD following exposure to ZnONPs (2 mg), UVB (150 mJ/cm^2^) and PT (100 µM). Values are presented as the mean ± SD (n = 3). *p < 0.05, control group versus treatment groups. ^#^*p* < 0.05, the UVB + ZnONPs groups versus the UVB + ZnONPs + PT groups

We next sought to determine whether UVB irradiation of mouse skin enhanced ZnONP-induced skin inflammation and to assess the effectiveness of topical PT treatment in the inhibition of acute skin inflammation. SKH-1 hairless mice were first administered a single dose of UVB radiation. Twenty minutes after radiation, 2 μg/cm^2^ ZnONPs alone or in combination with PT cream were applied to the mice, as demonstrated in Additional file 1: Figure 5A, B. The results indicated that skin thickness fold and skin redness were increased in mice exposed to UVB radiation and ZnONPs (Fig. 8A) (Additional file 1: Figure 5A, B). Moreover, after UVB irradiation and ZnONPs exposure, skin wrinkling and hyperplasia were observed by histological staining. Transepidermal water loss (TEWL, measured by standard evaporimetry), which reflects skin barrier function, was also increased in UVB irradiation- and ZnONP-exposed mice (Fig. 8B). In contrast, treatment with the topical PT cream (100 μM) following UVB radiation and ZnONPs exposure (Fig. 8C) decreased the skin thickness fold, skin redness and TEWL.

To confirm the previously identified in vitro toxicity mechanism, we next investigated the expression of the NLRP3 inflammasome proteins NLRP3, ASC, pro-caspase-1 and cleaved caspase-1 and the pyroptosis protein GSDMD in mice skin tissue. As shown in Fig. 8D–G, the expression of NLRP3, ASC, cleaved caspase-1 and GSDMD was increased in the ZnONP- and UVB-treated group but was significantly decreased in the PT cream-treated group. These data indicated that in an in vivo experiment, cotreatment with UVB radiation and ZnONPs induced acute skin damage, activated the NLRP3 inflammasome and induced skin pyroptotic cell death. Topical treatment with PT alleviated the severe skin inflammation and damage triggered by UVB radiation and ZnONPs exposure through the inhibition of NLRP3 inflammasome complex. The expression of ASC and cleaved-caspase-1 expression were significantly decrease, whereas the NLRP3 expression was only slightly decrease without statistical significance after the treatment of PT due to unknown reason. These findings corresponded with the in vitro experimental data.

## Discussion

**Fig. 9:**
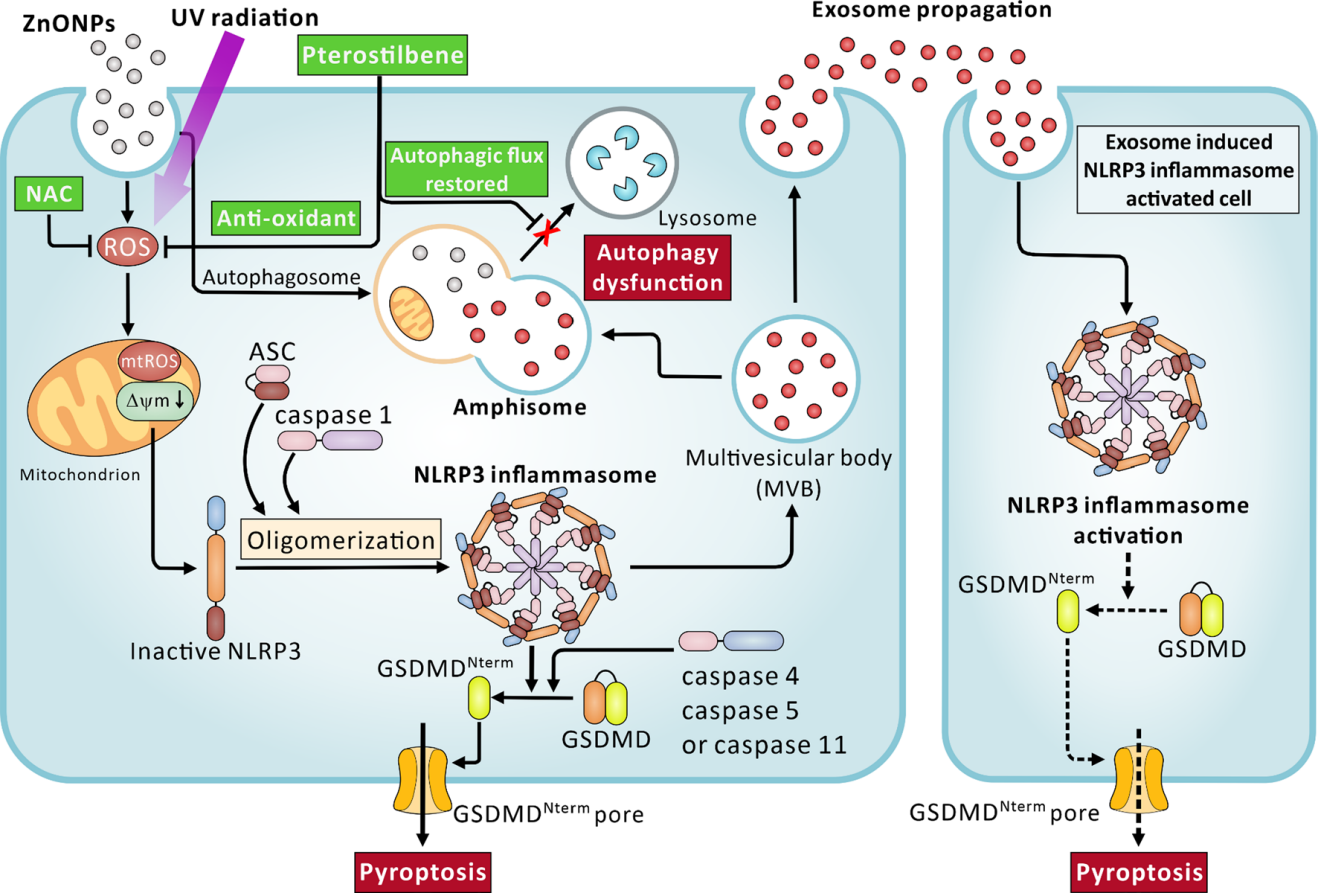
Schematic of the mechanism by which ZnONPs and UVB induce dermal toxicity and the inhibition mechanism of PT. Exposure to ZnONPs and UVB induced NLRP3 inflammasome-related pyroptosis, autophagy dysfunction and NLRP3 inflammasome loaded exosome secretion in keratinocytes. ZnONPs and UVB induced NLRP3 inflammasome, and pyroptosis through ROS generation and mitochondrial dysfunction. Moreover, ZnONPs also caused cell autophagy dysfunction (red cross), inhibited the fusion of NLPR3 complex protein loaded MVB and autophagosomes to form amphisome, which thereby inhibited the degradation of cargo in MVB and increased the amount of exosome-containing NLRP3 complex that propagated. In addition, PT protected HaCaT cells by attenuating ZnONP- and UVB-induced ROS generation and autophagy dysfunction

In this study, we first determined the effects of ZnONPs and UVB-induced keratinocytes damage that occurs through NLRP3 inflammasome-mediated pyroptosis, autophagy dysfunction, and exosome secretion. The major finding of this study is that the topical application of ZnONPs and UVB led to (I) significant skin surface damage, (II) enhanced generation of ROS, (III) mitochondrial damage and mtROS release and (IV) NLRP3 inflammasome activation, eventually causing pyroptotic cell death. In addition, ZnONPs exposure also caused (V) cellular autophagy dysfunction, which induced (VI) increased cell exosome release. We also demonstrated that PT attenuated skin damage by (I) exerting antioxidant effects and (II) restoring normal autophagic flux, resulting in lack of activation of the NLRP3 inflammasome-dependent pyroptosis cell death and consequently (III) decreased exosome propagation (Fig. 9).

The safety issue of the ZnONPs in cosmetics has been arguing in decades, although several studies indicated that ZnONPs might not penetrate skin and cause toxicity unless the skin barrier is impaired [4, 5]. In our results, we showed the combination of UVB and ZnONPs exposure can cause severe skin inflammation by ROS production. Many studies indicated the cytotoxicity of ZnONPs is often associated with its photocatalytic feature and the ROS generation as the major cause of nanotoxicity [12, 43]. Raghupathi et al. showed that ZnONPs under UV exposure can enhance the ROS production. such reactive species are superoxide anion (O_2_^−^), hydrogen peroxide (H_2_O_2_) and hydroxyl radical (OH·) [44]. To minimize the aggregation of nanoparticles, the ion shedding effect, semiconductor activity, and free radical formation, ZnONPs are commonly coated with various surface coatings, such as polystyrene, SiO_2_, a carboxyl group or an amine group [3, 45]. In the present study, we used amine-modified zinc oxide nanoparticles (NH_2_-ZnONPs), which is a positively charged nanoparticle widely used in research, with multiple applications such as UVB absorption, photocatalytic, and antimicrobial effects [46–48]. The different surface charged nanoparticle might have different modes of toxic action. For example, cationic nanoparticles might cause stronger mitochondrial damage and higher autophagic activation owing to their cation effect [49]. The surface charge also alters cellular uptake rate. Study showed that positively charged particles were generally easier taken up by cells than negatively charged particles [49]. It has also been demonstrated that cationic ZnONPs were more potent to induce toxicity than anion or non-ionic ZnONPs in THP-1 cells [50].

Studies reported cell can activated the innate immune response when exposed to ZnONPs [50, 51]. The NLRP3 inflammasome, an innate immune response protein complex, can be activated upon cellular infection or stress induced by PAMP, the so call pathogen-associated molecular patterns or DAMP, damage-associated molecular patterns. The NLRP3 inflammasome, a multiprotein platform, consists of a sensor (NLRP3), an adaptor (ASC) and an effector (caspase 1) [16]. Inflammasome activation is considered as a two-step process, including upregulation of NLRP3, ASC, caspase-1 and pro-IL-1β and following the recognition of an NLRP3 activator. NLRP3 is activated in microorganism infections as well as in sterile inflammation mediated by DAMPs and upon exposure to environmental irritants such as nanoparticles [14, 16]. The activated NLRP3 inflammasome can trigger the adaptor protein p62 to recognize exogenous stress-damaged organelles such as mitochondria and inflammasome-related proteins, leading to the induction of the autophagy pathway [52]. Recently, many different types of nanoparticles were shown to be foreign-derived DAMPs that can activate the NLRP3 inflammasome [14, 16, 37]. For instance, mesoporous silica nanoparticles (MSNs) trigger liver inflammation and hepatocyte pyroptosis via NLRP3 inflammasome activation [37]. Similar results were found with ZnO nanoparticles, which activated the NLRP3 inflammasome in A549 cells [3]. Our recent study revealed that the NLRP3 inflammasome was involved in hexavalent chromium-induced allergic contact dermatitis [33]. In this study, we further demonstrated that ZnONPs and UV radiation are NLRP3 activators that are able to induce the NLRP3 inflammasome during keratinocyte cytotoxicity and cause cellular inflammation.

Regarding the upstream mediators implicated in NLRP3 activation, mitochondrial dysfunction and the release of mtROS and mitochondrial DNA (mtDNA) into the cytosol are considered to be the key events [14, 16]. ROS as a byproduct of oxidative phosphorylation was produced continually by mitochondria, whereas under cellular stress situation, mtROS levels can be largely increased [14, 16, 53]. Many studies indicate that nanoparticles or pollutants have the ability to induce cells toxicity [37, 54]. For example, silver nanoparticles (AgNPs) has been reported to induce significant mitochondrial dysfunction and generate mtROS [55]. In this study, we demonstrated that ZnONPs and UVB led to significant mitochondrial dysfunction, including decreases in mitochondrial membrane potential and mtROS generation, thus causing NLRP3 inflammasome activation and pyroptosis (Figs. 3, 4). Application of the ROS scavenger NAC further confirmed that ROS played a crucial role in ZnONP- and UVB-activated NLRP3 inflammasome and pyroptosis (Fig. 5).

In addition to triggering inflammation, the NLRP3 inflammasome also leads to pyroptosis [16]. One important mediator of pyroptosis has recently been identified as GSDMD [36, 56]. The N-terminal cell death domain of GSDMD binds to phosphatidylinositol phosphates and phosphatidylserine in the cell membrane inner leaflet, oligomerizes and inserts into the plasma membrane, leading to the formation of a 10 to 14 nm pore containing 16 symmetrical protomers, and induces pyroptotic cell death [36, 56]. Another characteristic of GSDMD-dependent pyroptosis is the facilitating release of IL-1β and IL-18 via non-conventional secretion [57]. Recently, many nanoparticles have shown the ability to induce cellular pyroptosis, such as mesoporous silica nanoparticles (MSNs) and carbon nanotubes [16, 37]. MSN-induced ROS generation can activate NLRP3 inflammasome and trigger liver inflammation and hepatocyte pyroptosis [37]. Consistent with these results, we found that ZnONPs and UVB not only led to NLRP3 inflammasome activation but also induced pyroptotic cell death in keratinocytes, suggesting that skin damage induced by ZnONPs and UVB could be dependent on ROS generation and NLRP3 inflammasome-triggered pyroptosis.

To date, several plausible pathways related to nanomaterial-induced autophagy dysfunction have been reported. For example, our previous findings revealed that the internalized AgNPs through endocytosis accumulated in lysosomes or autophagosomes, which caused more lysosomal swelling, autophagy arrest and cell death [58]. Consistent with these results, we found that ZnONPs and UVB also induced autophagic dysfunction. As indicated in Fig. 6, accumulation of the autophagosome proteins LC3B and p62 was induced by ZnONPs and UVB, indicating autophagy dysfunction. Immunofluorescence staining also showed autophagosome (LC3B) and lysosome (LAMP-1) dislocation, implying the blockage of autophagy. The degradation of damaged mitochondria and the NLRP3 complex by autophagy prevents excess production of IL-1β mediated by the NLRP3 inflammasome [24]. Thus, ZnONP- and UVB-induced autophagy dysfunction contributed to NLRP3 inflammasome activation.

The effects of zinc ion generated by ZnONPs dissolution are also critical in ZnONPs toxicity [59]. In the present study, we did not investigate the cytotoxic effect of zinc ion released by ZnONPs. A previous study reported that ZnONPs could be taken up through cellular endocytosis, and dissolved in the intracellular endosome, with the accumulated cytosolic zinc ion leading to mitochondrial dysfunction, caspase activation, and cell apoptosis [59]. However, the precise mechanism of zinc ion–induced toxicity remains unclear. A recent study also indicated that autophagy dysfunction could be regulated by zinc ion, with intracellular zinc ion shedding in ZnONPs playing a crucial role. ZnONP-derived zinc ion accumulation affects lysosome–autophagosome fusion by disrupting the microtubule function and subsequent autophagy dysfunction [60]. Thus, the potential toxicity of lysosomal-autophagy dysfunction originating from ZnONPs-derived zinc ion is noteworthy and warrants further investigation.

Exosomes are a subtype of extracellular vesicles (EVs), which between 50 and 130 nm in diameter and are enriched in a set of molecular markers with an endosomal source [42, 61]. Exosomes are initially formed by the early endosomes that gradually mature into late endosomes, or multivesicular bodies (MVBs), these intermediate organelles are termed MVBs because of this morphology. During maturation, some endosomes undergo another membrane invagination and fission event that produces intermediate organelles characterized by numerous intraluminal vesicles. MVBs can fuse with the plasma membrane to release intraluminal vesicles into extracellular space, creating exosomes [26, 42]. Many studies have indicated that the release of exosomes, which are essentially the “garbage bags” of the cell, can promote the progression of Parkinson's disease and Alzheimer’s disease [41]. In addition, NLRP3 inflammasome-loaded exosome propagation has become an attractive research area [41]. A novel pyroptotic-phagocytic cascade has been shown to spread ASC and the NLRP3 inflammasome between cells, thus propagating the inflammasome [62]. In this study, we found that keratinocytes exposed to ZnONPs and UVB released exosomes loaded with NLRP3 inflammasome complexes. These kinds of exosomes can propagate and stimulate inflammasome activation in neighboring cells. Exosome biogenesis and autophagy have recently been proven to share similar molecular machinery, and these processes can be regulated by each other and play important roles in health and disease [34, 42]. Recent studies revealed that inhibition of autophagy may rescue exosome release from MVBs, that would otherwise be degraded [42, 63]. A study indicated that autophagosomes can fuse with MVBs to form amphisome and eventually fuse with lysosomes to recycle the cargo [42]. It has been demonstrated that autophagy impairment not only suppressed autophagic degradation of late endosomes but also promoted secretion of exosomes which caused migration, proliferation of hepatic stellate cells (HSCs) and promoted liver fibrosis [64]. Similarly, we found that exosomes release was markedly increased under the autophagy dysfunction. As showed in Fig. 7, increased number of exosomes containing inflammasome proteins were released under the exposure of ZnONPs and UVB and internalized by the other normal keratinocytes. When the autophagy inducer rapamycin was applied to restore the autophagic flux, the levels of inflammasome-related proteins released in exosomes significantly decreased, indicating that the blockade of autophagic flux by ZnONPs and UVB enhanced exosome release induced by malfunction of the autophagy recycling system (Fig. 7). Collectively, we demonstrate for the first time that ZnONPs and UVB induced inflammation could be regulated through exosome propagation through autophagy pathway.

We used PT as a therapeutic agent to attenuate ZnONP- and UVB-induced skin damage. Due to the hydrophobicity of PT, a simple emulsification method was used to increase the bioabsorption of PT in skin. Our data showed that PT treatment significantly decreased total ROS generation, mtROS and mitochondrial dysfunction caused by ZnONPs and UVB exposure. Similarly, PT treatment inhibited NLRP3 inflammasome activation and pyroptosis. We previously revealed the similar result that PT attenuates allergic contact dermatitis via inhibition of IL-1β-related NLRP3 inflammasome activation [33]. In addition to its antioxidant effect, PT reversed autophagy abnormalities by restoring normal autophagic flux. PT treatment lowered intracellular NLRP3 inflammasome protein levels and NLRP3 inflammasome-loaded exosome release. Another study also reported a similar pathway in which taurine, a sulfur-containing β-amino acid, improved arsenic-induced NASH by inhibiting autophagic inflammasome pathways [38]. More recently, we also proved that PT contributes to the prevention of renal fibrosis by attenuating NLRP3 inflammasome activation and inducing autophagy [35]. In summary, topical treatment with PT is a promising therapeutic approach that can attenuate nanoparticle- and UVB-induced skin damage through multiple inhibition mechanisms.

## Conclusion

The mechanisms and biomarkers of the toxicity of nanomaterials are currently topics of great interest in nanotoxicology. Our study reports a novel underlying molecular mechanism of ZnONP- and UVB induced skin damage that occurs through NLRP3 inflammasome-mediated pyroptosis, autophagy dysfunction and exosome secretion (Fig. 9). Furthermore, the results showed that PT attenuated ZnONP- and UVB-induced ROS generation and restored autophagy in HaCaT cells. An in vivo experiment also proved that ZnONP- and UVB-induced inflammation and pyroptosis in skin tissues was attenuated by PT. To the best of our knowledge, this is the first report investigating the protective effects of PT against skin damage by preventing cell pyroptosis through attenuating NLRP3 inflammasome activation and autophagy induction. However, subsequent studies are warranted to investigate the detailed interplay among the NLRP3 inflammasome, autophagy and exosome secretion pathways triggered by ZnONPs and UVB. A more profound understanding of the toxicity mechanisms of ZnONPs will facilitate the development of prevention and intervention policies against adverse consequences induced by ZnONPs or other metal and metal oxide nanomaterials.

## Methods

### Chemicals and reagents

Dulbecco’s modified Eagle’s medium (DMEM), penicillin, and streptomycin were purchased from Gibco BRL (Grand Island, NY, USA). Dimethyl sulfoxide (DMSO) was purchased from Sigma Chemical Co. (Poole, Dorset, UK). N-acetylcysteine (NAC) was obtained from Merck Chemical Co. (Darmstadt, Germany). Pterostilbene (PT) were provided by Dr. Chi-Tang Ho (Department of Food Science, Rutgers University, New Brunswick, NJ, USA). The synthesis method of PT was reported by Pettit et al. [65] and the purity of PT was determined by high-performance liquid chromatography (HPLC) to be greater than 96%.

### Preparation of pterostilbene cream

Pterostilbene cream was prepared by the simple emulsion method [66] to improve its absorption rate for topical treatment of the skin. First, 3 mg of PT was dissolved in 1 mL of olive oil, and then 0.5 mL of Span 20 (Sigma-Aldrich, #85544) and 2 mL of distilled water as the continuous phase were added and stirred for 20–30 min until the solution became a cream. The PT cream was prepared at 1 mg/mL (4 mM).

### Mice

SKH-1 hairless mice were a gift from Tzu-Kai Lin, M.D. (Department of Dermatology, College of Medicine, National Cheng Kung University, Taiwan). All animal experiments were followed the guidelines of animal center institute and were approved by the Institutional Animal Care and Use Committee of National Cheng Kung University, Taiwan (Approval No.: 106202). Male SKH-1 hairless mice (10–12 weeks of age) were acclimatized for 1 week before the start of experiments. Mice had free access to drinking water and standard rodent laboratory chow (No. 5001; Laboratory Rodent Diet, Texas, USA). Mice were housed five per cage at 12-h light/dark cycle and kept at 24 ± 2 °C.

### In vivo* experiment (Acute UVB irradiation)*

Mice were irradiated with UVB (acute minimal erythema dose of UVB in SKH-1 hairless mice, single exposure to 150 mJ/cm^2^) [31]. UVB irradiation was performed using a Crosslinker (UVP CL-1000). Twenty minutes after irradiation, the mice were topically treated with or without ZnONPs (2 mg/cm^2^) and then treated with or without PT cream (100 μM/10 μL) or control cream in the same area. The transepidermal water loss (TEWL) was measured at 24, 48 and 72 h after the treatment. Mice were sacrificed at the time points described in the results, the skin was immediately harvested for further analysis. Skin samples were first fixed with 4% paraformaldehyde in PBS, then dehydrated and paraffin-embedded for further histopathological analysis. The skin samples were stored at − 80 °C before protein analysis.

### Skin thickness measurement

Hematoxylin and eosin staining of paraffin-embedded skin samples was used to determine the epidermis thickness by microscopy then analysis with ImageJ software. Volume is expressed as micrometer in skin thickness.

### Cell culture

Human keratinocyte HaCaT cells were a gift from Professor Hamm-Ming Sheu (Department of Dermatology, College of Medicine, National Cheng Kung University, Taiwan). The cells were cultured in DMEM (Gibco, NY, USA) supplemented with 10% fetal bovine serum (Gibco, NY, USA), 5% CO_2_ at 37 ℃. HaCaT cells were first received a single UVB radiation (68 mJ/cm^2^) by using a Crosslinker (UVP CL-1000). After the irradiation, HaCaT cells were treated with ZnONPs under the indicated concentrations (10, 12.5 or 15 μg/mL) for 3, 24, 48 and 72 h. HaCaT cells were pre-treated with or without PT (1 or 2 μM) for 1 h, and then co-treated with ZnONPs.

### Cell viability assay

HaCaT cells were seeded in each of 12 wells (1 × 10^5^/well) and treated with different concentrations of PT (1 or 2 μM), ZnONPs (10, 12.5, or 15 μg/mL) and 68 mJ/cm^2^ UVB radiation for 24, 48 and 72 h. After exposure, the cells were trypsinized, centrifuged for 5 min at 100×*g*, and then resuspended in PBS. The cell suspension was diluted by mixing with 0.4% trypan blue. 20 μL of the trypan blue/cell 1:1 mixture was applied to a hemocytometer, and the unstained (viable)/stained (nonviable) cells were counted separately.

### ROS analysis

The cellular ROS generation was detected by using H_2_DCF-DA, (Sigma-Aldrich, #D6883). HaCaT cells were first treated with 20 μM H_2_DCF-DA, incubated at 37 °C for 30 min then washed with PBS. Ultimately, the cells were trypsinized and resuspended in PBS and analyzed by FACSCalibur flow cytometry (BD, San Jose, CA, USA). Ten thousand cells were collected per sample. Data was analyzed by FlowJo 7.6.1 software.

### Mitochondrial membrane potential (MMP) analysis

The level of mitochondrial membrane potential was estimated using the MitoTracker® Red CMXRos Probe (Invitrogen, M5712). After the treatment, cells were treated with 100 nM MitoTracker® Red CMXRos and incubated at 37 °C for 30 min and washed with PBS. The cells were trypsinized and resuspended in PBS and analyzed by FACSCalibur flow cytometry. Ten thousand cells were collected per sample. Data was analyzed by FlowJo 7.6.1 software.

### Mitochondrial superoxide analysis

Mitochondrial superoxide level was estimated by using the MitoSOX™ Red Probe (Invitrogen, MM36008). The cells were treated with 5 μM MitoSOX™ Red CMXRos, incubated at 37 °C for 30 min and washed with PBS. Ultimately, the cells were trypsinized and resuspended in PBS and analyzed by FACSCalibur flow cytometry. Ten thousand cells were collected per sample. Data was analyzed by FlowJo 7.6.1 software.

### LDH release analysis

HaCaT cells were exposed to PT (2 μM) for 1 h before treatment with 10 μg/mL ZnONPs and 68 mJ/cm^2^ UVB. After 24 h of exposure, the culture medium supernatants were collected. LDH levels in culture medium were estimated by using LDH Cytotoxicity Assay Kit (TaKaRa, MK401) according to the manufacturer’s protocol. The absorbance was read at 490 nm wavelength with a microplate reader (Thermo Fisher Scientific).

### Acridine orange (AO) staining

Acridine orange (AO) (Invitrogen, Carlsbad, CA, USA) was measured the number of acidic vesicular organelles (AVOs), such as autophagolysosomes, in cells. After the indicated treatments, cells were incubated with AO (1 μg/mL) for 20 min at 37 °C in the dark and then washed with PBS. Ultimately, cells were trypsinized and resuspended in PBS and analyzed by FACSCalibur flow cytometry. Ten thousand cells were collected per sample. Data was analyzed by FlowJo 7.6.1 software.

### Immunofluorescence staining and confocal microscopy analysis

HaCaT cells were seeded on coverslip-bottom dishes. After treatment, the cells were fixed with 4% paraformaldehyde for 10 min, washed 3 times with PBS and treated with 0.5% Triton X-100 (Sigma, T8787) to penetrate the cell membranes. The samples were stained with the indicated primary antibody for 1 h at 37 °C and incubated with Alexa Fluor 488- or Alexa Fluor 594-conjugated secondary antibody (1:200) for 1 h at 37 °C. The nuclei were stained by DAPI staining. Images were obtained with an LSM 780 confocal microscope (Zeiss, Germany) (Instrument Development Center, NCKU) and analyzed using the Zeiss confocal software ZEN 2010.

### Exosome isolation

HaCaT cells were cultured in UltraCULTURE™ Serum-free Cell Culture Medium (Lonza, #12-725F) to prevent contamination with EVs from fetal bovine serum. HaCaT cells were exposed to PT (2 μM) for 1 h before treatment with 10 μg/mL ZnONPs and 68 mJ/cm^2^ UVB. After 24 h of exposure, the culture supernatants medium was collected and the exosome were isolated via differential centrifugation. Briefly, the supernatants were centrifuged at 300×*g* for 5 min to eliminate the cells, at 3000×*g* for 10 min to eliminate cell debris, and at 10,000×*g* for 30 min to eliminate microvesicles. The supernatants were eventually centrifuged at 100,000×*g* for 120 min using a Beckman Optima L-100XP Ultracentrifuge with an SW28 rotor (Beckman) to separate the soluble compounds from the exosomes, which were washed once in 0.2 μm filtered particle free PBS.

### NanoSight

The concentration and size distribution of exosome samples were estimated by using nanoparticle tracking analysis (NTA), as previously described [24]. Exosomes were diluted and resuspended in 500–1000 μL of PBS. Approximately 300 μL sample was loaded into the sample chamber of an LM10 unit (NanoSight, Amesbury, UK) by using a syringe. Sample were analyzed with NTA 2.3 software (NanoSight).

### Transwell experiment

To demonstrate the process of exosome propagation between cells, a coculture system with Transwells was used to mimic the cellular milieu by treating HaCaT cells with 10 μg/mL ZnONPs and 68 mJ/cm^2^ UVB on the apical surface; the detailed protocol was described previously [67]. A total of 4 × 10^4^ HaCaT cells were cultured for 24 h in the upper chamber of the Transwell with 0.4-μm pores (Corning #3413). The HaCaT cells were labeled with DioC18 dye (5 μg/mL; Invitrogen, D275) at 37 °C for 30 min. After staining, the cells were washed three times with PBS to remove excess dye. Then, the HaCaT cells were cocultured with unstained HaCaT cells that were seeded at 4 × 10^5^ cells in the lower compartment of the system. The HaCaT cells were treated with 10 μg/mL ZnONPs and 68 mJ/cm^2^ UVB and with or without 20 nM rapamycin and 50 nM chloroquine. Exosomes from DioC18-labeled HaCaT cells were released and internalized by the HaCaT cells in the lower compartment, which was observed using fluorescence microscopy and quantified by flow cytometry analysis.

### Western blot analysis

For western blotting, cell or tissue extracts were made by homogenization in lysis buffer. About 20 μg of protein (determined by BCA assay) was boiled with Laemmli buffer. Whole-protein extracts were separated on 6–15% SDS–polyacrylamide gels and transferred to polyvinylidene difluoride membranes (Merck Millipore, Darmstadt, Germany). After 1 h blocking procedure, the membranes were probed with primary antibodies in 1:1000 dilution. The antibodies for anti-NLRP3, ASC, caspase-4, caspase-5, pro-GSDMD, cleaved-GSDMD, LC3-II, flotillin-1, HSP70 and GAPDH antibodies were purchased from Cell Signaling (Beverly, MA, USA); anti-pro-caspase-1, cleaved-caspase-1 antibodies were purchased from Proteintech (Rosemont, IL 60018, USA); anti-Lamp-1, CD63 antibodies were purchased from Novus (Centennial CO 80112, USA); anti-TSG-101, p62 antibodies were purchased from Abcam Inc. (Cambridge, MA, USA). After the hybridization, membrane was washed with 1× TBST, and then probed with HRP-conjugated anti-mouse (Biolegend, San Diego, CA, USA) or anti-rabbit secondary antibodies (Jackson ImmunoResearch Lab Inc., West Grove, PA, USA) in 1:10,000 dilution. The immunoreactive proteins were visualized with Immobilon Western chemiluminescence HRP substrate (Merck, USA) and BioMax LightFilm (Eastman Kodak Company, New Heaven, CT, USA) according to the manufacturer’s instructions.

### Statistical analysis

SigmaPlot 10.0 (Systat Software, Inc., USA) was used to analyze the data and the results were presented as mean ± standard deviations (SD). Comparisons of means were performed by Student’s *t* test. *p* value < 0.05 was considered statistically significant difference.

## Supplementary Information

**Additional file 1.** Supplementary information of  Experimental Section and Figures.

## Data Availability

All data generated or analysed during this study are included in this published article (and its supplementary information file).

## Abbreviations

ZnONPs: Zinc oxide nanoparticles
UV: Ultra violet
TEWL: Transepidermal water loss
PT: Pterostilbene
ROS: Reactive oxygen species
mtROS: Mitochondrial reactive oxygen species
GSDMD: Gasdermin D
TEM: Transmission electron microscopy
DLS: Dynamic light scattering
AO: Acridine orange
CQ: Chloroquine
mtDNA: Mitochondrial DNA
PAMPs: Pathogen-associated molecular patterns
DAMPs: Damage-associated molecular patterns
AgNPs: Silver nanoparticles
EVs: Extracellular vesicles
MVBs: Multivesicular bodies
