# Supplementary material for: Skin damage induced by zinc oxide nanoparticles combined with UVB is mediated by activating cell pyroptosis via the NLRP3 inflammasome–autophagy–exosomal pathway
Source: Part Fibre Toxicol. 2022 Jan 5;19:2. doi: 10.1186/s12989-021-00443-w (PMC8729117; doi:10.1186/s12989-021-00443-w)
Supplement: Supplementary file 1 — Additional file 1. Supplementary information of Experimental Section and Figures. [file 12989_2021_443_MOESM1_ESM.docx]

**Supplementary Material**

**Skin Damage Induced by Zinc Oxide Nanoparticles Combined with UVB is Mediated by Activating Cell Pyroptosis via the NLRP3 Inflammasome-Autophagy-Exosomal Pathway**

Yu-Ying Chen ^a^, Yu-Hsuan Lee ^b^, Bour-Jr Wang ^c, d^, Rong-Jane Chen ^e, *^, Ying-Jan Wang ^a, f, *^

**Supporting Information for Experimental Section**

***Preparation and Characterization of ZnONPs*:**

This study used amine-modified zinc oxide nanoparticles (NH_2_-ZnONPs). First, 3.35 mM zinc acetate dihydrate (Zn(CH_3_COO)_2_(H_2_O)) in 31.25 ml ethanol was mixed with potassium hydroxide (KOH) in 16.25 ml methanol and stirred at 60°C. Then, KOH was added dropwise into the mixture and reacted for 1.5 hours until the solution became turbid. The precipitate was collected by centrifugation at 10,000 rpm for 10 min, and then the pellet (ZnONPs) was washed twice with ethanol. Next, 0.25 ml APTES ((3-aminopropyl) triethoxysilane), 0.05 ml 25wt% ammonia and 0.5 ml distilled water were added to the ZnONPs solution and stirred for 20 hours at RT. The precipitate was collected by centrifugation at 10,000 rpm for 15 min. The pellet was resuspended in distilled water, and the resulting solution was the NH_2_-ZnONP solution.

***Transmission Electron Microscopy Analysis*:**

HaCaT cells were exposed to PT (2 μM) for 1 h before treatment with 10 μg/ml ZnONPs and 68 mJ/cm^2^ UVB. After 24 hours of exposure, the cells were collected and fixed with 2% glutaraldehyde and 1% osmium tetroxide, rinsed in 100 mM sodium phosphate buffer (pH 7.2), dehydrated in ethanol and embedded in Epon (Sigma, 45347). Ultrathin sections of the HaCaT cells were collected on formvar-coated grids, stained with 10% uranyl acetate and 1% lead citrate, and then examined with a JEOL JEM-140 transmission electron microscope (JEOL, Japan) operated at 120 kV.

***sh-RNA knockdown assay:***

Caspase-1 shRNAs were obtained from the National RNA Interference Core Facility (Institute of Molecular Biology/Genomic Research Centre, Academia Sinica, Taipei, Taiwan). The human library is referred to as TRC-Hs 1.0. The individual clone was identified as shRNA TRCN0000003503. The stable shRNA knockdown cells were following the protocol of National RNA Interference Core Facility (http://rnai.genmed.sinica.edu.tw/index). Briefly, after seeding cells, 2-3 MOI (multiplicity of infection) of lentivirus was added in growth media containing polybrene. After 24 hours infection, media were replaced with fresh media. Then the cells were selected by added 2-5 μg/ml puromycin at least 48 hours. The stable shRNA knockdown cell line was use for following experiment.

**Supplementary data,
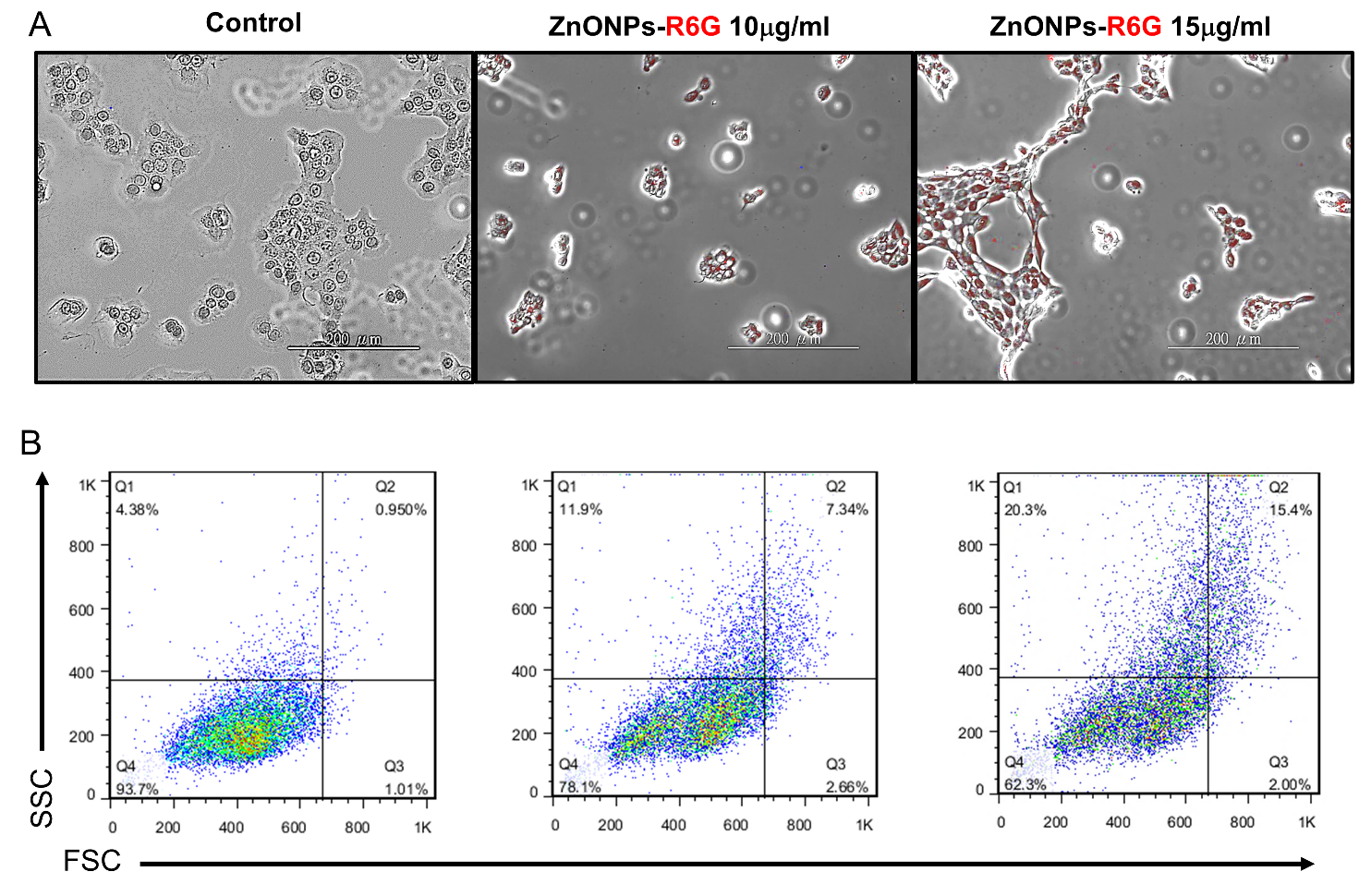
 Figure 1.** Cellular uptake of ZnONPs. (A) The cellular uptake of ZnONPs was measured by using R6G-ZnONPs (10 and 15 μg/ml) after a 24-h incubation with HaCaT cells. (B) The FSC and SSC increased as the dose of ZnONPs increased (10 and 15 μg/ml) after a 24-h incubation with HaCaT cells.

**Supplementary data,
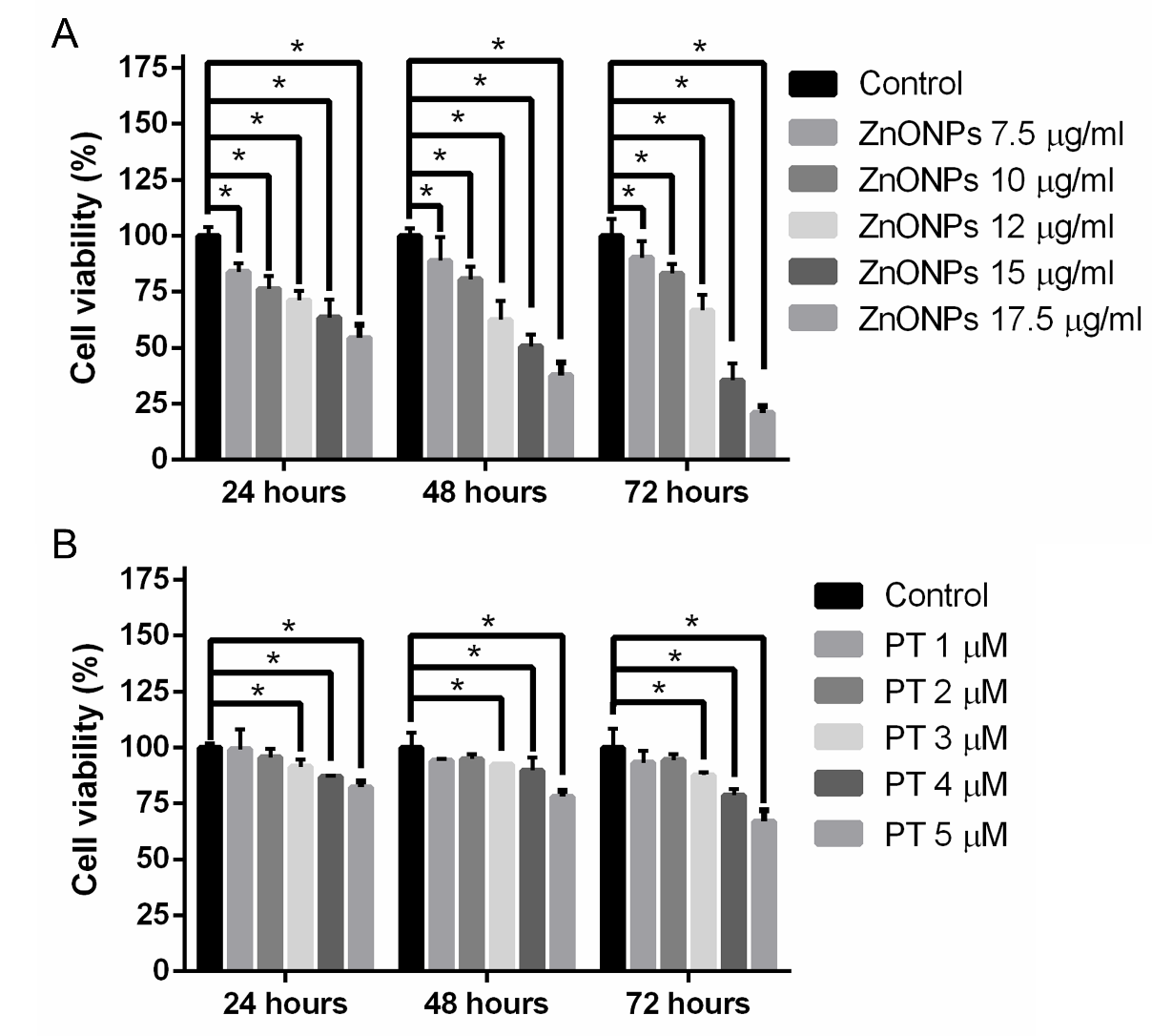
Figure 2.** Viability of HaCaT cells treated with ZnONPs and PT. (A) Cell viability assay showing the dose-dependent cytotoxicity of alone treatment with ZnONPs (0-17.5 μg/ml). (B) Cell viability assay showing the dose-dependent cytotoxicity of alone treatment with PT (0-5 μM). Values are presented as the mean ± SD (n = 3). *p < 0.05, control group versus treatment groups.

**
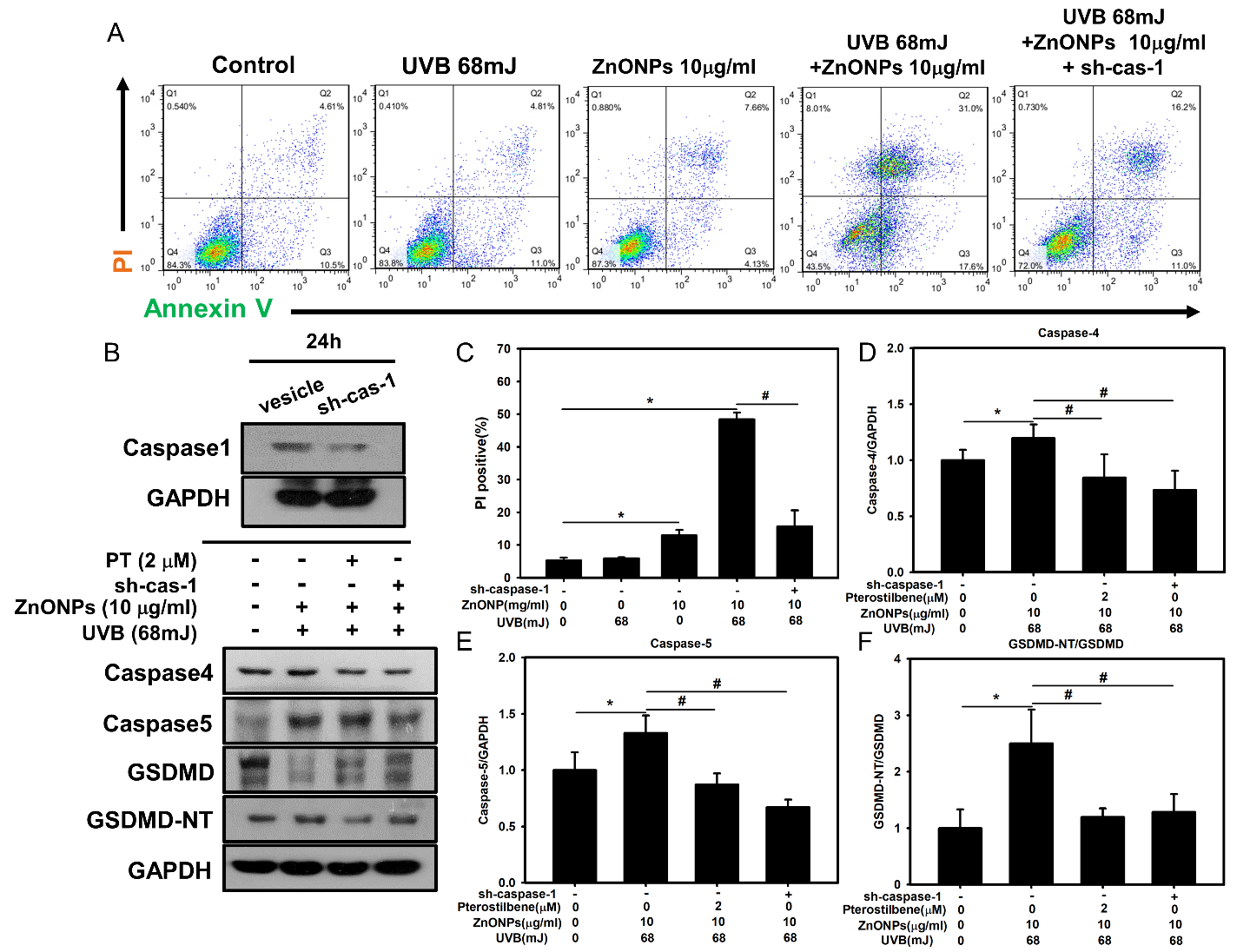
Supplementary data, Figure 3.** Pyroptosis induced by ZnONPs and UVB in HaCaT cells is NLRP3 inflammasome-dependent. (A)(C) Annexin V and PI were employed to examine the effect of sh-caspase-1 against ZnONP-induced keratinocyte pyroptosis via flow cytometry. The percentage of PI positive cells indicate the Q1+Q2 regions. (B)(D-F) Western blot analysis of the effects of caspase-1 silencing on the ZnONP-induced pyroptosis proteins caspase 4, caspase 5, GSDMD and cleaved GSDMD-NT in HaCaT cells. Nonsilencing shRNA was used as a control. Values are presented as the mean ± SD (n = 3). *p < 0.05, the UVB+ZnONPs groups versus the control group. # p < 0.05, the UVB+ZnONPs groups versus the UVB+ZnONPs+sh-cas-1 groups.

**
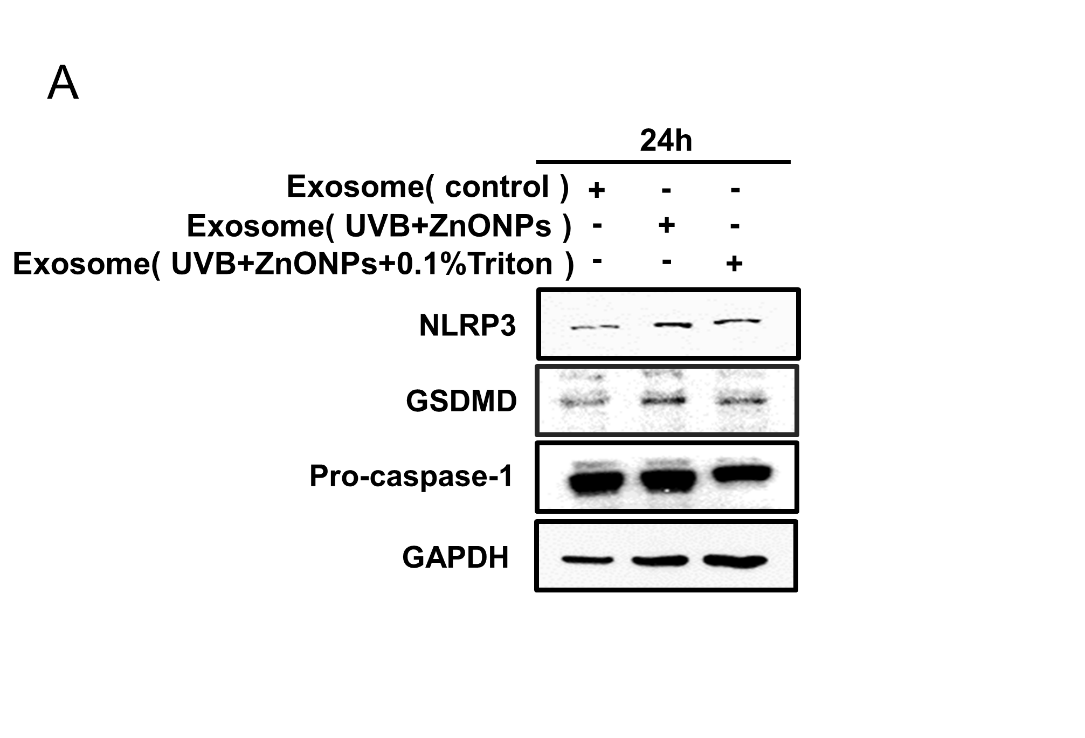
**

**Supplementary data, Figure 4.** NLRP3 inflammasome-loaded exosomes mediate the transmission of cell inflammation. (A) Western blot analysis of NLRP3, pro-caspase-1 and GSDMD expression in HaCaT cells after treatment with exosomes. When Triton X-100 (0.1%) was used to permeabilize the exosomes, the exosome-mediated transmission of cell inflammation decreased.


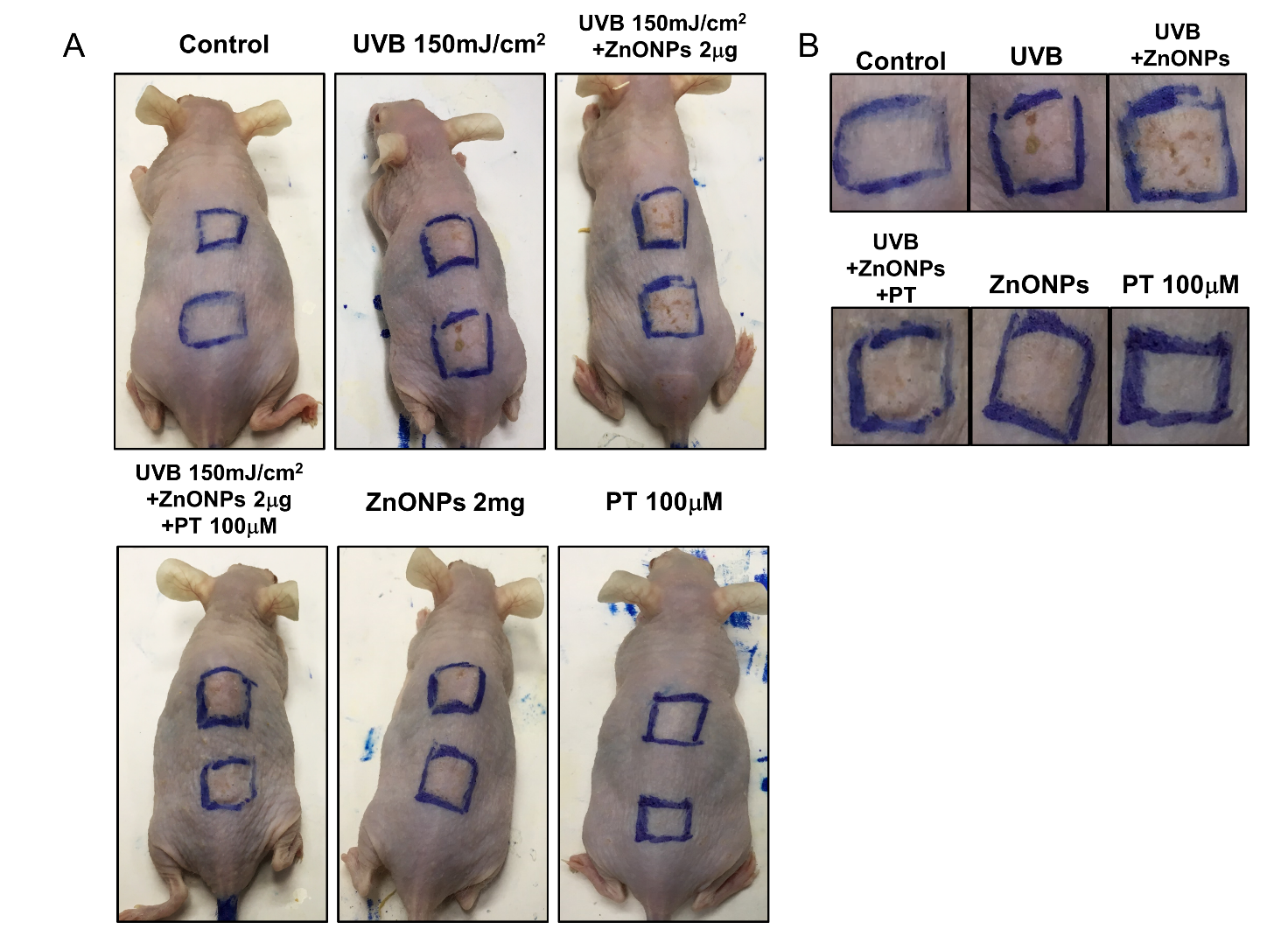


**Supplementary data, Figure 5.** Effects of pterostilbene protects the skin against pro-inflammatory induced by ZnONPs and UVB exposure. (A)(B) Pictures showed skin redness induced by UVB (150mJ/cm^2^), ZnONPs (2mg) and PT (100μM) alone or in combination.
